# Neuro-molecular characterization of fish cleaning interactions

**DOI:** 10.1038/s41598-022-12363-6

**Published:** 2022-05-19

**Authors:** S. Ramírez-Calero, J. R. Paula, E. Otjacques, R. Rosa, T. Ravasi, C. Schunter

**Affiliations:** 1 The Swire Institute of Marine Science, School of Biological Sciences, The University of Hong Kong, Pokfulam Rd, Hong Kong SAR, China; 2 MARE—Marine and Environmental Sciences Centre, Laboratório Marítimo da Guia, Faculdade de Ciências da Universidade de Lisboa, Av. Nossa Senhora do Cabo, 939, 2750-374 Cascais, Portugal; 3 Pacific Biosciences Research Center, Kewalo Marine Laboratory, University of Hawai’i at Manoa, Honolulu, HI USA; 4 Marine Climate Change Unit, Okinawa Institute of Science and Technology Graduate University, 1919-1 Tancha, Onna-son, Okinawa, 904-0495 Japan; 5 Australian Research Council Centre of Excellence for Coral Reef Studies, James Cook University, Townsville, QLD 4811 Australia

**Keywords:** Behavioural genetics, Gene expression, Behavioural ecology, Molecular ecology, Learning and memory, Social behaviour

## Abstract

Coral reef fish exhibit a large variety of behaviours crucial for fitness and survival. The cleaner wrasse *Labroides dimidiatus* displays cognitive abilities during interspecific interactions by providing services of ectoparasite cleaning, thus serving as a good example to understand the processes of complex social behaviour. However, little is known about the molecular underpinnings of cooperative behaviour between *L. dimidiatus* and a potential client fish (*Acanthurus leucosternon*). Therefore, we investigated the molecular mechanisms in three regions of the brain (Fore-, Mid-, and Hindbrain) during the interaction of these fishes. Here we show, using transcriptomics, that most of the transcriptional response in both species was regulated in the Hindbrain and Forebrain regions and that the interacting behaviour responses of *L. dimidiatus* involved immediate early gene alteration, dopaminergic and glutamatergic pathways, the expression of neurohormones (such as isotocin) and steroids (e.g. progesterone and estrogen). In contrast, in the client, fewer molecular alterations were found, mostly involving pituitary hormone responses. The particular pathways found suggested synaptic plasticity, learning and memory processes in the cleaner wrasse, while the client indicated stress relief.

## Introduction

Social behaviour allows species to establish biological relations through intra- and interspecific interactions. These relationships prompt species to generate social mechanisms to survive (e.g. detect predators), reproduce (e.g. courtship) and thrive in nature (e.g. territoriality, living in groups^1^). Indeed, social behaviour is an ability that promotes responses to specific situations (i.e. competition for shelter or food), including biotic factors and their physical environment^2^. This ability to respond to social stimuli can be regulated to optimize their relationships with conspecifics and other species, allowing them to perform more effectively in nature^1^. At present, the study of social behaviour and its mechanisms have been centred on understanding the capacity to regulate and change social relationships (social plasticity) that can enhance and promote survival^3,4^.

Studies on this behaviour have focused on the genetic, epigenetic, endocrine and neural mechanisms underlying social behavioural responses^1,5,6^. For example, the evolution of social phenotypes and transcriptomic signatures in mice, sticklebacks and honey bees, have elucidated the mechanisms regulated during the response to social challenges (e.g. territory intrusion)^7,8^. One well-studied group of genes is called immediate early genes (IEGs) and is used to detect early neural activation as indicators of adaptive plasticity and learning processes^9,10^. Other groups of genes suitable for understanding social responses (e.g. cooperation and aggression) are genes regulated in molecular pathways related with the neuron system (e.g. Dopaminergic pathway), or with the transduction of signals (stimuli) in the brain (e.g. MAPK signalling pathway)^11–13^. These pathways are modulated during the interactions or communications between individuals, and social behavioural phenotypes can therefore be linked to particular gene expression patterns^12,14^. While many studies focus on major social challenges (e.g. territory defense, cooperation, dominance), little is known about neuro-molecular responses of other key social interactions such as marine cleaning mutualisms. Cleaning mutualism is one of the most important interactions between coral reef fishes as it involves the removal of ectoparasites from the skin of cooperative hosts and establishing long-term social interactions that are key to maintaining the biodiversity of the ecosystem^15–17^. Therefore, to further understand the gene regulation of this type of social interaction, organisms displaying well-developed social systems and sophisticated cognitive abilities are essential to investigate the functional molecular basis of cleaning mutualisms^1,3,5,18^.

The coral reef bluestreak cleaner wrasse *Labroides dimidiatus* is one of the most remarkable examples of mutualistic cleaning behaviour in marine species. It is widely known for enhancing fish biodiversity in local communities due to its important role in cleaning ectoparasites from the skin of hosts and for its complex social behaviour^19,20^. Studies on this species as a model for behaviour, have focused on neural physiological responses in its interaction with other species and how social situations such as establishing social bonds modulate their behaviour^15,21–23^. For example, neurotransmitters dopamine, serotonin and arginine vasotocin (AVT)^24^ influence the motivation of cleaners to engage in interactions^25^ and are induced during social stress^26^. These monoaminergic hormones also play a role in the regulation of the cleaning service in this cleaner wrasse and its willingness to interact, thus modifying the capacity of individuals to react to new social scenarios (i.e. presence of new clients^27^). These monoamines can also be altered after prolonged exposure to different environmental conditions (such as high CO_2_), revealing a disruption of the cleaner’s perception, ability to negotiate and motivation to interact^28^. These observations suggest an involvement of specific neurohormones in the interaction behaviour of *L. dimidiatus*, but the molecular mechanisms implicated in this behaviour remain unknown.

Other social aspects of cleaner wrasse behaviour such as recognition or learning have been shown to involve the neuropeptides arginine vasotocin (AVT) and isotocin (IT) in different brain regions^23^. For instance, AVT alteration in the cerebellum or Hindbrain (HB) affects *L. dimidiatus* motivation to switch from clients to conspecifics revealing the cerebellum as a candidate area driving decision-making and memory processes^24^. High levels of AVT in the Forebrain (FB) also resulted in a delay in assortative learning for the cleaner wrasse when discriminating two tasks^29^. Furthermore, high levels of isotocin (IT) in cleaner wrasse FB are associated with conspecific support (i.e. positive physical contact) thus reducing anxiety^30^. The focus on different brain regions for the study of interaction behaviour is necessary to determine the different parts of the brain responsible for the behavioural aspects and three brain regions of teleosts are commonly studied when analysing social behaviour including the Forebrain (FB), Midbrain (MB) and Hindbrain (HB)^30–33^. By studying the brain regions separately and performing targeted studies on candidate neurotransmitters or hormones, key molecular processes can be linked with organisms’ social behaviour. Several studies have been conducted at behavioural and neurobiological scales (using either pharmacological or quantitative methods), but little is known about the molecular mechanisms mediating this essential interaction behaviour.

In this study, using transcriptomics, we evaluate the molecular signatures during the interaction behaviour of *L. dimidiatus* with its client fish the powder-blue surgeonfish *Acanthurus leucosternon,* across the Forebrain (FB), Midbrain (MB) and Hindbrain (HB) regions*.* We selected these brain areas to assess the transcription of candidate neurohormones and neuropeptides during cleaning interactions. We analysed whole-genome differential gene expression patterns by comparing cleaners and clients after social interactions against individuals without interaction (control). We expect to detect changes in gene expression of candidate neurohormones dopamine and serotonin and neuropeptides such as AVT and IT in different brain regions when *L. dimidiatus* engages in social interactions. Furthermore, we also investigate the gene expression patterns related to memory and learning processes as cleaner wrasses are known for their remarkable cognitive abilities and regularly use learning and memory to improve the outcome of their cleaning interactions^34,35^. Finally, identifying fundamental molecular processes altered in the interaction of these two species is key to understanding mutualistic cleaning behaviour and elucidating mechanisms of social plasticity during interspecific cleaning interactions.

## Results

### Behavioural analysis

In the interaction condition every pair (6 replicates) of *L. dimidiatus* and *A. leucosternon* engaged in cleaning interactions. On average, each pair engaged in 43 ± 17.5 interactions, with an average duration of 13 ± 3.6 s, corresponding to a proportion of time spent interacting of 13 ± 6.6% out of the 40 min of behavioural trials. Of these interactions, on average, 75 ± 13.7% were initiated by the cleaner. The cleaner fish’ dishonesty was answered with an average of 3 ± 2.8 client jolts and 2 ± 1.9 chases. Lastly, cleaners used tactile stimulation for reconciliation on average 3 ± 3.4 times. In the control, both *L. dimidiatus* and *A. leucosternon* were swimming around the tank without any display of abnormal behaviour or stress. All behavioural data can be found in Supplementary Table S1b.

### De novo transcriptome assembly

The obtained final de novo transcriptome assemblies are the first references for both *L. dimidiatus* and *A. leucosternon* and resulted in 114,687 and 123,839 coding transcripts respectively (NCBI accession: GJED00000000 and GJGS00000000). Values of N50 indicated that at least half of the transcripts had a length of 1813 and 2827 bases, respectively. For *L. dimidiatus* and *A. leucosternon*, a total of 26,380 and 30,770 transcripts were annotated using the Swissprot database, respectively. In addition, a total of 8379 and 6438 transcripts were annotated using the *Danio rerio* reference, and 28,988 transcripts were annotated only for *L. dimidiatus* using *Labrus bergylta* database. Further metrics and assembly steps can be found in Supplementary Tables S2 and S3.

### Gene expression analyses

#### *Labroides dimidiatus*

**Figure 1:**
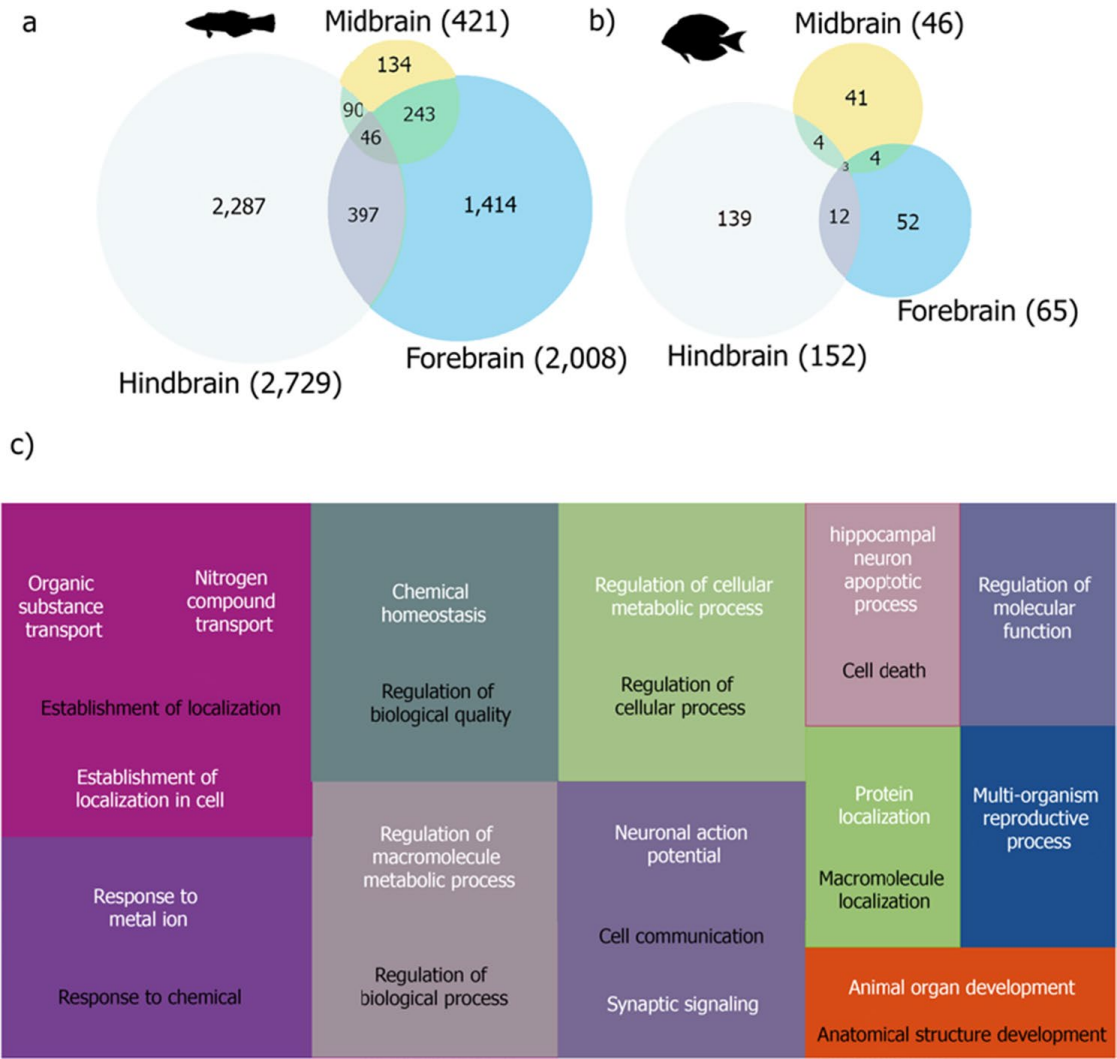
Number of differentially expressed genes between control and interaction for each brain region and the common overlap of (**a**) *Labroides dimidiatus* and (**b**) *Acanthurus leucosternon*. Numbers in brackets represent the total differential expressed genes found in each brain region. (**c**) Gene Ontology treemap for *L. dimidiatus* representing the commonly enriched functions across the three brain regions when interacting with a client. Boxes with the same colour correspond to the upper-hierarchy GO-term, and its title is found in the middle of each box. For *A. leucosternon* no common enriched functions were found across the three brain regions. Description of the enriched functions for both species can be found in Supplementary Tables S5–S10.

We evaluated the gene expression differences between individuals from the control, which had no interaction, against individuals from the interaction condition. 46 commonly differentially expressed genes (DEGs) exhibiting functions such as dendrite cytoplasm, mRNA binding, sodium ion transmembrane, transporter activity, ATP binding, positive regulation of dendritic spine morphogenesis (Fig. 1c; Supplementary Fig. S1, Table S4). When analysing differential gene expression for each of the three brain regions separately, we found that the Hindbrain (HB) exhibited the highest number of differential gene expression among the brain regions (2728 DEGs, Fig. 1a), followed by the Forebrain (FB; 1414 DEGs) and the Midbrain (MB; 421 DEGs). In the HB, 1370 significantly enriched functions were found, including negative regulation of neuron apoptotic process, synaptic cleft, AMPA glutamate receptor activity, long-term memory, sensory perception of sound, and a total of 14 enriched functions related to behaviour such as motor behaviour, grooming behaviour, behavioural fear response (Supplementary Table S7, Fig. S3). In the FB, 980 biological processes were related to behaviour such as signal transduction, calmodulin binding, social behaviour, locomotory exploration behaviour, and vocalization behaviour, among others (Supplementary Table S5, Fig. S1). Finally, for the MB, functional enrichment only resulted in 357 significant functions, including nuclear-transcribed mRNA catabolic process, nonsense-mediated decay, neuronal action potential and sodium ion transmembrane transport (Supplementary Table S6, Fig. S2). Biological functions regarding behaviour in this brain region were related to behavioural response to pain, locomotory behaviour, male courtship behaviour, maternal behaviour and behavioural defence response (Supplementary Table S6).

#### *Acanthurus leucosternon*

For *A. leucosternon*, the total number of DEGs was lower in comparison with *L. dimidiatus*. Only three common differentially expressed genes were found across the three brain regions, which are RNA/RNP complex-1-interacting phosphatase (DUS11), ATP-dependent DNA helicase PIF1 (PIF1) and a third gene for which no annotation was obtained. However, when analysing differential gene expression in each brain region separately, we found 139 DEGs in the HB and nine enriched in functions such as DNA metabolic process, DNA repair, biological regulation and protein binding (Supplementary Table S10). For the FB, 52 DEGs were detected with six enriched molecular functions in signalling receptor activator activity, receptor regulator activity, hormone activity, and two biological functions: gas and oxygen transport (Supplementary Table S8). Finally, 41 DEGs were found in the MB, and only Rab protein signal transduction was significantly enriched (Supplementary Table S9). Like *L. dimidiatus*, the HB exhibited the highest number of DEGs in the comparison of control vs interaction (Fig. 1a,b).

**Figure 2:**
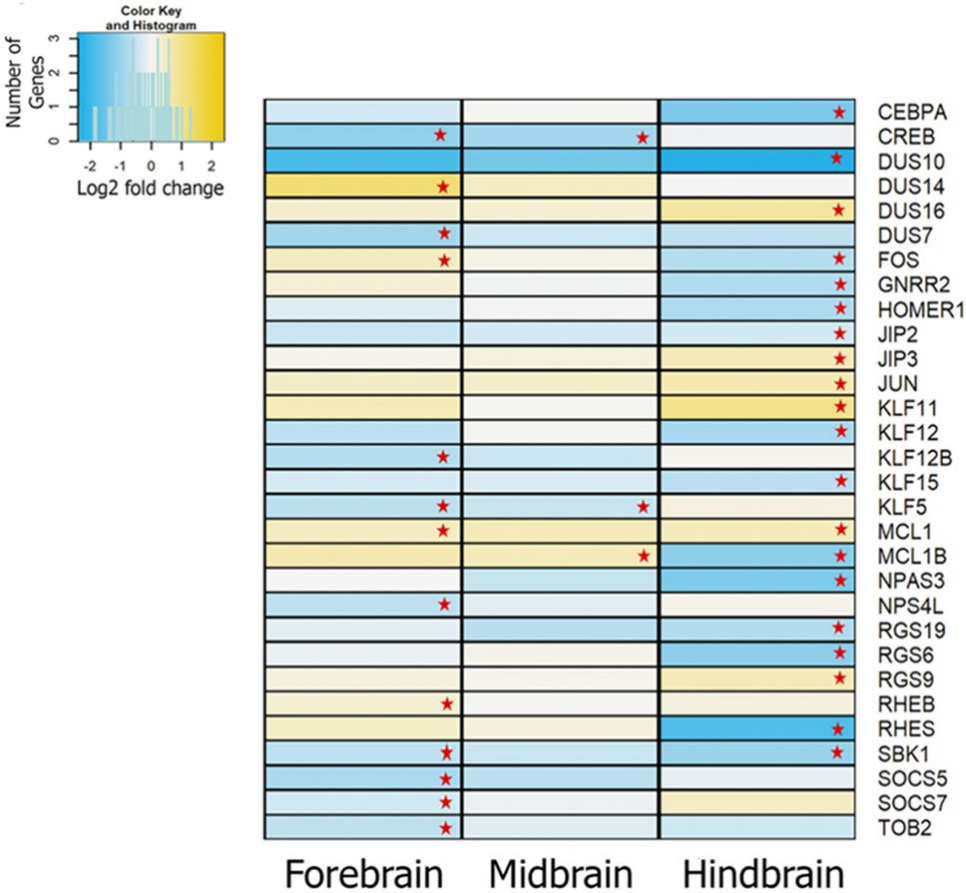
Comparative differential gene expression patterns of Immediate Early Genes between the regions of the brain of *L. dimidiatus*. Red stars represent significance. The legend indicates the reference values of log2fold changes for each DEG in the figure.

Evidence of significant differential expression of Immediate Early Genes (IEG) was found in *L. dimidiatus* with 31 differentially expressed IEGs in the three brain regions (Fig. 2). However, more differentially expressed genes were found in the HB region (20) and the FB region (16) (Fig. 2). Many of the functions of these genes are involved in signalling (i.e. RHEB, RGS6/9 and 19), transcription factor activation (i.e. KLF5/11) and plasticity (i.e. NPAS4L). Differential expression of genes associated with learning processes, memory and neural development was also observed. For instance, genes *c-fos* (FOS) and SBK1 in the FB and HB, being SBK1 downregulated in both regions, while *c-fos* in the HB only. Moreover, genes KLF11 and JUN were differentially expressed in the HB region (Fig. 2, Supplementary Table S11). Finally, for *A. leucosternon*, the only IEG differentially expressed found in our dataset was JUN in the HB, hence found differentially expressed in both studied species.

**Figure 3:**
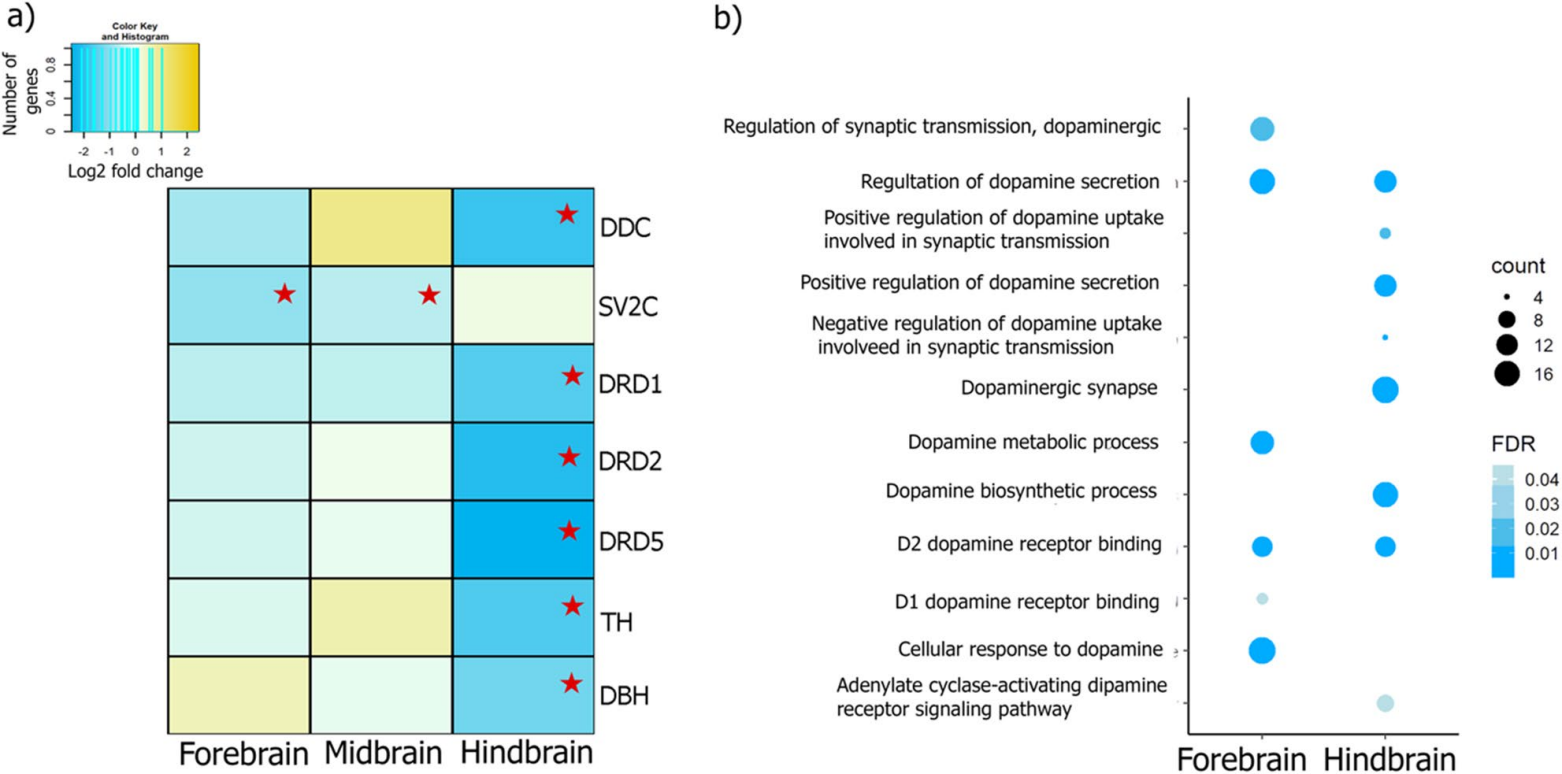
(**a**) Dopaminergic pathway differential gene expression in the three brain regions of *L. dimidiatus*. The colours presented correspond to log2fold changes. The red star represents significance. The legend indicates the reference values of log2fold changes for each DEG in the figure. (**b**) Functional enrichment of Gene Ontology (GO) terms related to Dopamine activity in *L. dimidiatus* in the Fore and Hindbrain. No enrichment was found for the Midbrain region. The size of the circles is proportional to the number of genes observed within each GO category, and the colour of the circles is proportional to the significance (FDR value).

Additional groups of genes differentially expressed during the interaction were found. For instance, Dopamine pathway genes such as Dopamine receptors DRD1, 2, 5, Tyrosine 3-monooxygenase (TH), Dopa Decarboxylase (DDC) and Dopamine beta-hydroxylase (DOPO) were differentially expressed in the HB region of *L. dimidiatus* (Fig. 3a). These genes were downregulated during the interaction condition, and enriched functions such as the regulation of dopamine secretion and D2 dopamine receptor binding were shared between the FB and HB, as well as hormones receptors of progesterone in FB, while estrogen and IT in HB (Supplementary Table S12). Furthermore, Dopamine biosynthesis processes, Dopaminergic pathway, adenylate cyclase-activating dopamine receptor signalling pathway and the regulation of dopamine secretion were specific for the HB, while the regulation of synaptic transmission, dopamine metabolic process, D1 dopamine receptor binding, and the cellular response to dopamine were enriched functions only in the FB for *L. dimidiatus* (Fig. 3b, Fig. S4). Finally, no differential expression of these genes was found in the MB region.

**Figure 4:**
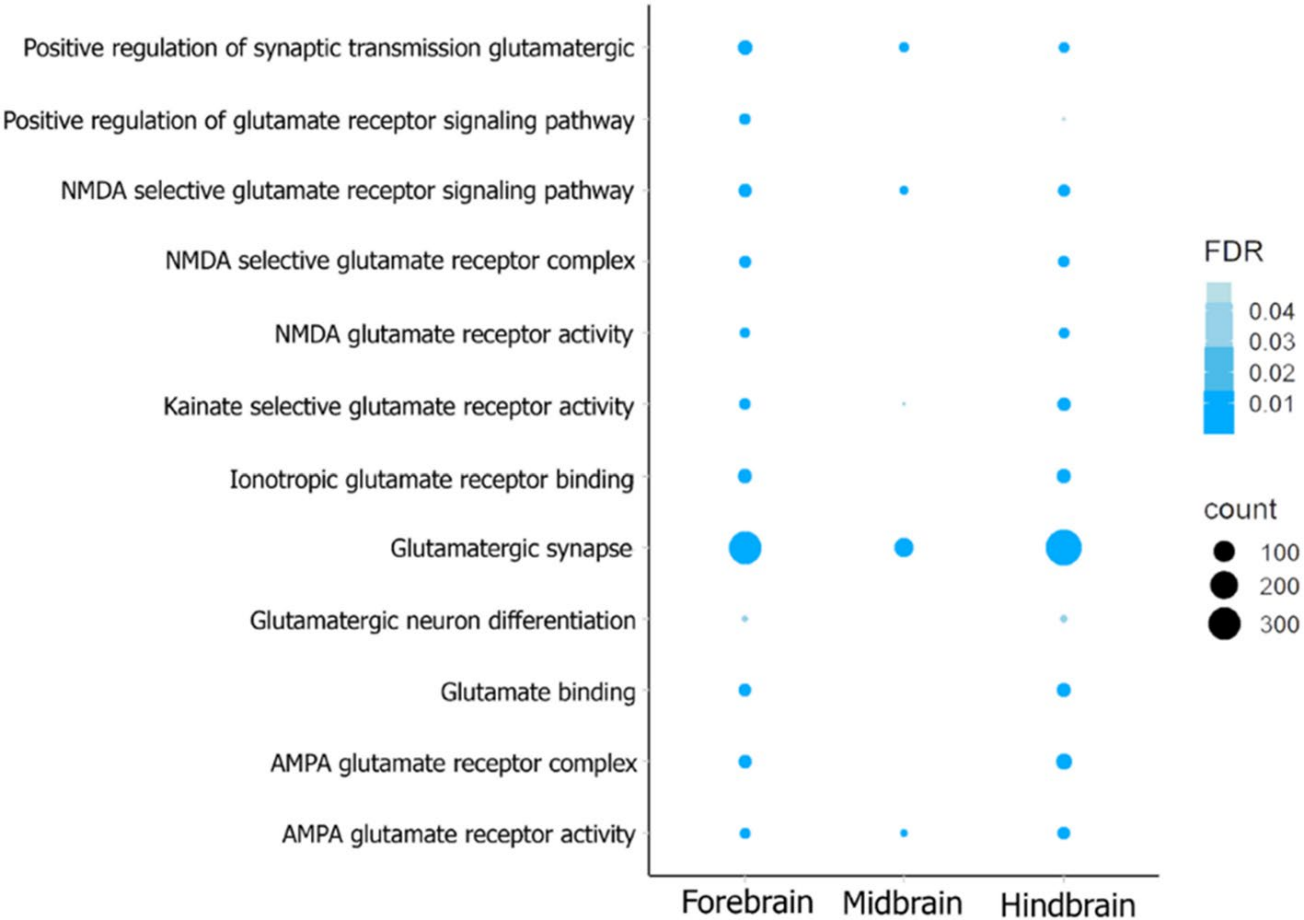
Functional enrichment of Gene Ontology (GO) terms related to the Glutamatergic synapse pathway in *L. dimidiatus* individuals in the Fore, Mid and Hindbrain. The enrichments are based on differentially expressed genes of the comparison control vs interaction. The size of the circles is proportional to the number of genes observed within each GO category, and the colour of the circles is proportional to the significance (FDR value).

Several differentially expressed genes (DEGs) were also found for *L. dimidiatus* related to the glutamatergic synapse pathway (Fig. 4, Supplementary Table S13, Fig. S5). Many enrichments related to this pathway were exhibited in the brain of interacting *L. dimidiatus*, such as positive regulation of synaptic transmission glutamatergic, NMDA selective glutamate receptor signalling pathway, kainate selective glutamate receptor activity and AMPA glutamate receptor activity, among others with enrichments in the three regions of the brain (Fig. 4). Interestingly, more functions were shared by the FB and HB, such as AMPA glutamate receptor complex, ionotropic glutamate receptor binding, NMDA glutamate receptor activity, glutamatergic neuron differentiation, glutamate binding and NMDA selective glutamate receptor complex (Fig. 4). These functions stem from a total of 72 DEGs in the brain of *L. dimidiatus*, playing a role in the glutamatergic synapse processes (Supplementary Table S13). The differentially expressed genes within this pathway also followed our previous general pattern in which the HB region reports a higher number of significant DEGs (48), followed by the FB (46) and the MB (16) (Supplementary Table S13). In particular, most of the ionotropic receptors (Supplementary Table S13) were differentially expressed in the HB and FB except for GRIA3 and GRIA4, which were also differentially expressed in the MB. These receptors exhibited a downregulation pattern in all three brain regions when interacting with another species. In contrast, metabotropic receptors were differentially expressed in HB and FB during interaction condition (Supplementary Table S13); however, main receptors GRM1, 5 and 7 showed upregulation patterns in these two brain regions, while GRM3 and 4 were downregulated at HB.

Only two DEGs related to the glutamate pathway were found in *A. leucosternon*: Glutamate carboxylase (DCE1) and Glutamate receptor ionotropic (GRID2). However, hormonal responses were enriched in the brain of *A. leucosternon* during the interaction condition (Supplementary Table S14). This was in concordance with the enrichment terms found in the FB region of this fish (Hormone activity, Supplementary Table S14). In particular, pituitary hormones such as Somatotropin (SOMA), Prolactin (PRL), Somatolactin (SOML2), Gonadotropins (GTHB1 and 2), Thyrotropin (TSHB) and Glycoproteins (GLHA) were differentially expressed and downregulated in the FB during the interaction condition, but highly expressed during the control (Fig. S6). Further differentially expressed genes were related to functions related to calcium-binding activity, oxygen transport and signalling were upregulated during the interaction in the FB (i.e. CHP2, FCRL5, PTPRF, ADA1B, MICU3, Supplementary Table S14).

## Discussion

Social stimulus during the interaction affected both fishes’ transcription regulation, and the larger number of altered molecular processes were found in the cleaner wrasses. We observed a change in gene expression of functions related to behaviour in *L. dimidiatus* (*e.g.* locomotory exploration behaviour) while interacting with the client. The genes included dopaminergic pathway genes, glutamatergic receptors (ionotropic and metabotropic), and immediate early genes (IEGs). The specific transcription found for *L. dimidiatus* suggests the activation of synaptic neurotransmission that can lead to mechanisms of memory consolidation in teleost fishes through glutamate^36^, and reward-learning processes driven by the expression of dopamine receptors and IEGs^1,31^. In cleaner wrasses, previous pharmacological and quantitative studies identified several neuromodulators of cleaning interactions, such as arginine vasotocin (AVT, lower cleaning motivation and reduced cooperation)^23,24,29^, cortisol^37^ (mediation of cooperation and cheating), and serotonin (enhanced motivation to interact)^25,34^. In our study, genes involved in dopamine, isotocin (IT), progesterone, estrogen and glutamate pathways, and several IEGs were differentially expressed. Hence, our results illustrate additional regulations across different brain areas at the transcriptional level when cleaner fish are exposed to the presence of a new client and engage in cleaning interactions. As for *A. leucosternon*, the small number of genes with altered expression observed, shows lower transcriptional activity of genes regulating interaction behaviour in this species, suggesting that clients don’t require large neurobiological reconfigurations to engage in cleaning interactions.

The transcriptional patterns in both interacting species were found mainly in the Forebrain (FB) and Hindbrain (HB), revealing the neural areas where behavioural modulation of cleaning interaction occurs. The HB (cerebellum, medulla oblongata and rhombomeres^32^) is in charge of motor activity and autonomic responses (i.e. eye retraction). This brain region is known to control major cognitive abilities of spatial learning and classical conditioning, such as the ability to learn from preceding events, usually by dopamine metabolites turnovers^38,39^. The large transcriptional changes exhibited for *L. dimidiatus* could result from a cascade of social stimulus processing underlined mainly by the downregulation of dopamine receptors 1 and 2 (D1/2), which were mostly differentially expressed in the HB. We also observed that the cleaner fish initiated 75% of cleaner-client interactions, implying a higher motivation to interact. Dopamine was previously described as a neuromodulator of cleaning interactions as pharmacological manipulation of the dopaminergic synapse with a D1 receptor antagonist led to increased motivation to engage in cleaning interactions^36^. Moreover, de Abreu et al.^38^, in a study with a similar experimental setting (cleaner fish paired with a client to interact in an aquarium), cleaner fish had a reduced dopamine concentration in the FB and MB following cleaning interactions^38^. Interestingly, we found that the gene SV2C, known for its regulatory role in dopamine release^40^, was downregulated in both FB and MB but not HB, suggesting that the downregulation of this gene it’s a plausible cause for the reduction of dopamine concentration observed in de Abreu et al.^38^. Consequently, our results, combined with previous studies, strongly corroborate the classification of dopamine as a major neuromodulator of cleaning interactions (mainly through a signal reduction).

During the interaction, we also found differential expression of the glutamatergic pathway, its ionotropic and metabotropic receptors and several voltage-dependent calcium channel subunits in the FB and HB of *L. dimidiatus*. Motivated behaviour, memory and decision-making have previously been shown to develop in the FB region in teleost fish^32^. For instance, the elevated expression of glutamate receptors in zebrafish has been associated with memories to respond to electric shocks^41^, motivated behaviour and learning^42^ while its distribution defines neural connectivity and plasticity during development^43^. Further evidence from a knifefish (*Apteronotus leptorhynchus)* suggests that the contribution of glutamate receptors in learning and memory is dependent on their distribution across the FB and HB in adults^44^. A link between glutamate and calcium channels with memory and learning under social situations has also previously been established using rodents^45^. Consequently, changes in gene expression patterns of the glutamate pathway suggest a contribution to synaptic plasticity, learning and memory in *L. dimidiatus*, especially for its concomitant expression with voltage-dependent calcium channel subunits^46^. For the cleaner wrasse learning and memory are fundamental for maximize the output of cleaning interactions, through partner prioritization, response to dishonesty and partner control mechanisms crucial for future encounters^4,21,47^. Nevertheless, the transcription of calcium channels and glutamate receptors with the social interactions of the cleaner wrasse has not been described before. Therefore, we propose the gene expression patterns of the glutamate pathway and calcium channel subunits to be one of the main molecular mechanisms underlining the interaction and contributing to cognitive functions of memory, learning and synaptic plasticity in the FB and HB regions of the cleaner wrasse.

Additional mechanisms were also identified in *L. dimidiatus* during the interaction through the differential expression of progesterone, estrogen and isotocin. Nevertheless, the expression of both progesterone and estrogen receptors in other fishes such as *A. burtoni* has led to the regulation of behavioural plasticity, and the evaluation of rewarding stimulus between dominant and subordinate males^10,48^ as well as the modulation of dominant behaviour in female zebrafish^9^. We found both estrogen and progesterone receptors downregulated during the interaction of the cleaner wrasse with its client, which could suggest a reduction in dominant behaviour. This regulation might be a response to partner control mechanisms imposed by the client to the cleaner to avoid cheating, such as chasing or jolting^49,50^. In addition to this, neuropeptides arginine vasotocin (AVT) and isotocin (IT) are known to regulate cooperative behaviour in teleost fish with expression in the hippocampus (FB)^51^ and HB^24^. High levels of IT can increase submission rates of *L. dimidiatus* in social groups and regulate its social sensitivity^38^. Both sex hormones and neuropeptides such as IT are involved in social behaviour responses to new challenges^51^. Even though, we did not find a differential gene expression of AVT as expected, the downregulated expression for IT, progesterone and estrogen receptors, may suggest the manifestation of a submissive response of *L. dimidiatus* when approaching new clients and in response to their partner control mechanisms.

To understand how changes in gene expression occur rapidly immediate early genes (IEGs), which are neurological activity markers, can indicate rapid shifts in behavioural states^1^. During the interaction, *L. dimidiatus* differentially expressed IEGs across brain regions during the interaction revealing an important activation of transcription factors and their participation in the transduction of signals. For instance, the expression of *c-fos* has been reported in the hippocampus region (FB) of cichlid fish *A. burtoni* during social and territorial interactions of males, indicating regulation of memory, spatial processing and social recognition of non-dominant males^33^. Thus, the upregulation of *c-fos* in the FB of *L. dimidiatus* may suggest similar neural mechanisms activated to recognize and interact with its clients. In addition, CREB regulatory factor (CREB), another IEG, is critical for the consolidation of memory^52^, and it was differentially expressed in the cleaner wrasse. Studies examining the molecular mechanisms of learning and memory in feeding and consolidation of new habits using mandarin fish (*Siniperca chuatsi*) also revealed differential expression of this gene when fish learn to eat dead prey after a training period^53^. Therefore, the differential expression of CREB may suggest the activation of downstream processes of memory in the FB and MB when interacting with clients, which correspond to areas where associative learning and memory occur in teleost fishes^32^. Differential expression of IEGs may therefore contribute to social recognition, memory and learning, which are important processes for the cleaner wrasse to choose how to approach each client with the choice whether to clean, cheat or provide tactile stimulation^34^.

The molecular signatures found underlying the interaction behaviour in the cleaner wrasse were not evident in its client, the powder-blue surgeonfish. In the client, we observed differential expression of several hormones in the FB such as Somatotropin releasing-hormone (SOMA), Prolactin (PRL), Somatolactin (SOML2), Pro-opiomelanocortin (POMC), among others, suggesting the transcriptional activity of the hypothalamic-pituitary-thyroid (HPT) axis^54^. While HPT in teleost fishes is mainly known for controlling development and growth through the secretion of these hormones^54–56^, they also play a role in several behavioural aspects of fishes^57,58^. For instance, SOMA and PRL can regulate locomotion, feeding behaviour and cognitive functions in zebrafish^41,59^ and aggression in rainbow trout^60^. SOML2 regulate stress in Atlantic cod and flounder^55^ while POMC control levels of cortisol (CRH) leading to stress and food intake disruption in rainbow trout^61^. Accordingly, as the HPT hormones found in our client fish had a significantly lower expression during the interaction, this may hint to a change in the behaviour towards the cleaner wrasse and a decrease in stress in *A. leucosternon*. We cannot rule out that some of the gene expression patterns arose from taking the client from a group environment and placing it into a tank alone, but this did not increase stress levels on the transcriptional or behavioural levels. In fact, *L. dimidiatus* is well-known for providing stress relief to clients by lowering cortisol levels through physical contact (known as tactile stimulation)^17^, and our behavioural trials support this since cleaners engaged on average in 3 ± 3.4 tactile stimulation events during the interactions. Consequently, since physical contact from *L. dimidiatus* can reduce stress in clients and downregulate HPT hormones related to stress was observed during the interaction, our results suggest stress-relief behaviour at the molecular level in the client fish.

In conclusion, differences in gene expression patterns in both fishes were noticeable, being *L. dimidiatus* the species with large transcriptional reprogramming compared to *A. leucosternon*. During their interaction, transcriptional regulation of genes involved in social behaviour was altered in expression for *L. dimidiatus* mainly in the FB and HB regions. A possible increase in cleaners’ willingness to interact and promote tactile stimulation was observed by the transcription of genes related to the dopamine pathway. The transcription of the glutamatergic synapse pathway and voltage-dependent calcium channel subunits across the brain regions of *L. dimidiatus* suggested a contribution to learning, memory and synaptic plasticity processes. Although we did not account for finer brain regions, we propose that the concomitant expression patterns of glutamate and calcium channel subunits deserve particular attention to explore the consolidation of cognitive abilities in cleaner wrasses. Furthermore, the downregulated gene expression of isotocin, estrogen and progesterone may reveal a reduction of dominant behaviour to approach clients. Finally, *c-fos* and CREB highlight additional molecular mechanisms associated with social recognition, memory and learning in *L. dimidiatus*. In contrast, in the client *A. leucosternon*, the major molecular signal corresponded to a decreased transcription of hormones related to the HPT axis, indicating stress reduction during the interaction. Our results highlight the transcriptional patterns underlying the mutualistic cleaning behaviour in the cleaner wrasse *L. dimidiatus* driving synaptic plasticity, learning and memory. These processes are crucial for *L. dimidiatus* to enhance the outcome of cleaning interactions key to biodiversity in coral reef ecosystems.

## Materials and methods

### Experimental setup

All experiments reported below were performed in accordance with relevant guidelines and regulations of the Animal Research: Reporting In Vivo Experiments (ARRIVE guidelines^62^).

**Figure 5:**
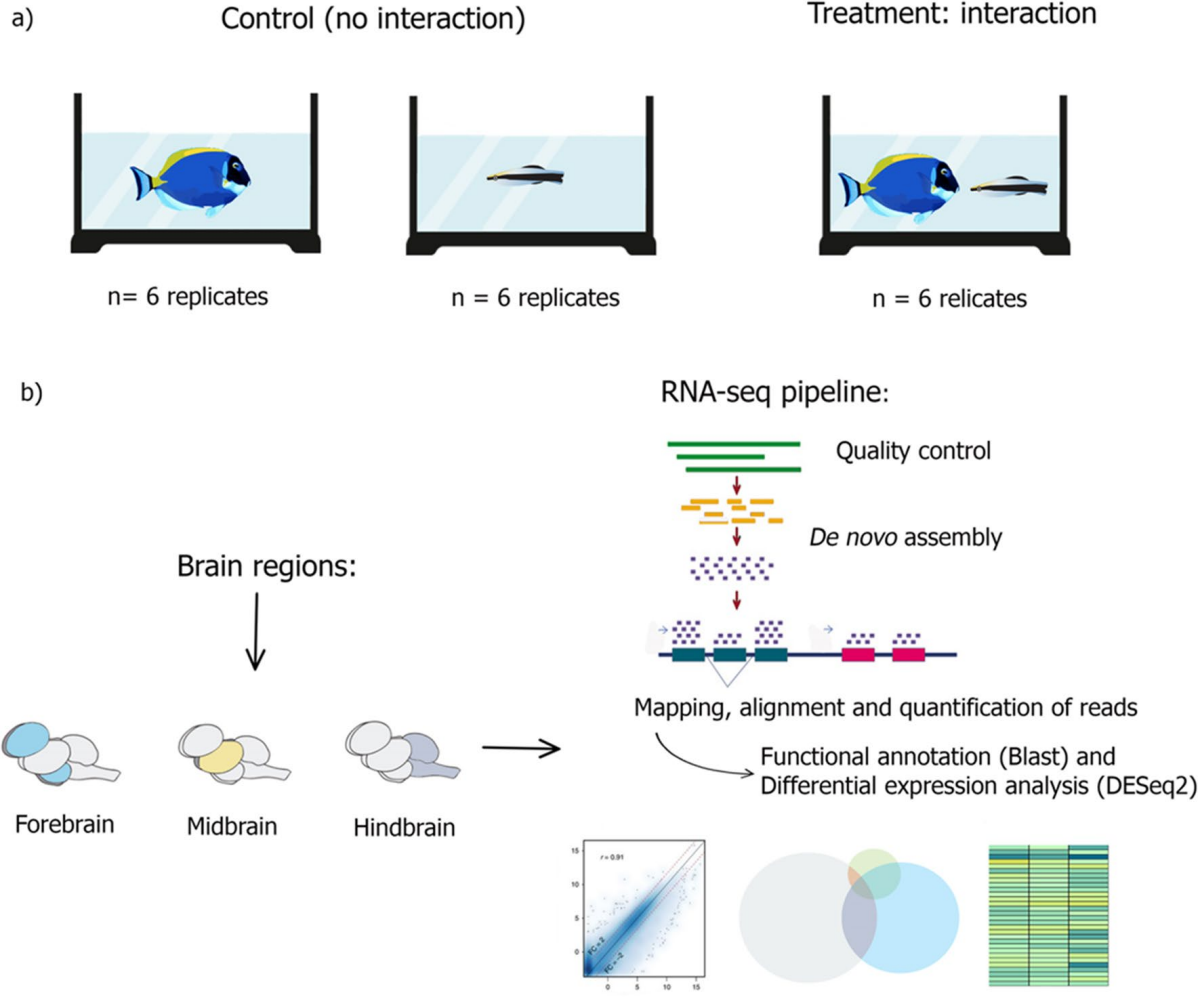
(**a**) Experimental design in which *Labroides dimidiatus* (N = 12) and *Acanthurus leucosternon* (N = 12) were kept separately (control: no interaction) or allowed to interact (condition: interaction) (DGAV—Permit 2018-05-23-010275). 40 min of videos were recorded. (**b**) Brain-regions dissections (Forebrain, Midbrain and Hindbrain) for each species, and RNA-seq pipeline including de novo transcriptome assembly, differential gene expression and functional analysis. Further details of the individuals used can be found at Supplementary Table S1a.

To test for the molecular mechanisms involved in the interaction between two fish species, 12 female adult individuals of *L. dimidiatus* and 12 female adults of *A. leucosternon* were collected from the wild in the Maldive Islands and transported by TMC-Iberia to the aquatic facilities of Laboratório Marítimo da Guia in Cascais, Portugal (Fig. 5, Supplementary Table S1a). We selected a common Acanthurid species as a client since they are one of the most frequent hosts for *Labroides* species in coral reefs^21^. We also selected female individuals to be consistent with past studies using *L. dimidiatus* as a model species and because gene functions may differ between sexes and can blur the analysis of molecular signals^63^. Fishes were habituated for 28 days to laboratory conditions. Water parameters were monitored daily and automatically controlled using an aquarium computer (Profilux 3.1 N GHL, Germany). Seawater conditions were kept similar to their capture site (salinity = 35 ± 0.5, temperature 29 ± 1 °C, pH 8.10 ± 0.05). Each cleaner fish was kept separately in individual tanks (20L) to avoid aggression as they are highly territorial animals. In contrast, surgeonfish (*A. leucosternon*) were held in groups of three individuals in 20 L tanks. Fish were fed *ad libitum* once per day. Each experimental tank had a flow-through aquatic system in which alkalinity levels, dissolved carbon and pH were strictly maintained. Natural seawater was pumped from the sea to a storage tank of 5 m^3^ and then filtered and UV-irradiated with a Vecton V2 300 Sterilizer before reaching the experimental tanks. Experimental tanks were kept under a photoperiod of 12 h/12 h (light/dark cycle). Ammonia and nitrate levels were checked daily using colourimetric tests and always kept below detectable levels (Salifert Profi Test, Netherlands). Seawater temperature was regulated using chillers (Frimar, Fernando Ribeiro Lda, Portugal) and underwater heaters 300 W, (TMC-Iberia, Portugal). Salinity was daily monitored with a V2 refractometer (TMC-Iberia, Portugal), and pH and temperature with a VWR pH 1100H pH meter (Avantor, US).

Behavioural tests started after 28 days of laboratory acclimation. The tests were performed in observation tanks (40 L) set in an isolated observation room. The fish were either placed into a tank with (i) no-interaction (control) or (ii) interaction (cleaner-client pair). In the no-interaction treatment, cleaners or clients were kept alone in the observation tank (control), while for the interaction treatment, pairs composed of one cleaner and one client were kept together in the observation tank, allowing them to have close contact (Fig. 5). Their interactions were filmed for 40 min since it is documented that this is the time frame in which neurohormones, and peptides are activated during cleaner interactions^23^. At the end of the observation period, each fish was immediately euthanized by severing the spine, body length was measured, and three separated regions of the brain were immediately dissected out (Fig. 5b): Forebrain (diencephalon and secondary prosencephalon), Midbrain (tectum opticum, torus semicircularis and tegmentum), and Hindbrain (cerebellum and medulla oblongata), as behaviour is regulated differently in these regions of the brain in teleost fishes^23^. Finally, tissues were placed in a tube, snap-frozen and kept at − 80 °C for further processing.

### Behavioural video analyses

Behavioural analysis was performed in both treatments. For no-interaction individuals (control), we looked for abnormal behaviour and stress such as erratic movement, secession of swimming or aggressive postures. No abnormal or stress like events were found. For the interaction treatment individuals, we analysed cleaning behaviour according to Paula, J. R. et al.^28^. Cleaning behaviour was grouped into two categories, (i) cleaning interactions and motivation and, (ii) interaction quality^25^. To characterise cleaning interactions and motivation, we measured the number of interactions (i.e. close body inspection and removal of damaged tissue or scales including inspection allowing posture of the client), the number of interactions initiated by both cleaners and client, as well as the number of posing displays the client conducted to attract the cleaner. Interaction quality was determined by the duration of interactions, the number of client jolts (conspicuous signals that indicate cheating or dishonesty by the cleaner^49^), the number of times clients were chased by the cleaners to initiate interactions, and the number and duration of tactile stimulation events (touches with pectoral fins that reduce stress levels and prolong interaction duration^17,64^. All behavioural videos were analysed using the event-logging software “Boris”^65^ using the behavioural catalogue defined in Supplementary Table S1b with both the cleaner fish and the client as focal objects, and further information can be found on Supplementary Table S1c.

### RNA extraction and transcriptome assembly

For RNA extractions 300 μl of RTL Buffer was added to the frozen tissue with several sterile silicon beads for tissue homogenization in a Tissuelyzer (Qiagen) for 30 s at maximum speed to then follow the RNAeasy Mini Kit protocol including a DNase I treatment (Qiagen). The resulting RNA was tested for quality on an Agilent Bioanalyzer and all samples met quality standard of RNA Integrity Number (RIN) > 8. mRNA-focused sequencing libraries were designed with Illumina TruSeq v3 kits and sequenced for 150 bp paired-end reads on an Ilumina Hiseq4000 at the King Abdullah University of Science and Technology corelab facility.

In order to assess the molecular basis to species interactions, on average 31.4 million raw reads for *L. dimidiatus* and 33.8 million for *A. leucosternon* were processed following a bioinformatic pipeline (Supplementary Table S2a,b). Quality was examined using FastQC v. 0.11.9^66^, reads were trimmed, and adapters were removed to avoid the presence of poor-quality sequences in our de novo transcriptome assembly. For this, we used Trimmomatic v.0.36^67^ with parameters as follows: ILLUMINACLIP: TruSeq3-PE.fa:2:30:10 LEADING:4 TRAILING:3 SLIDINGWINDOW:4:15 MINLEN:40. For both species, there is currently no genome reference available, hence a de novo transcriptome assembly was constructed for both species separately after several tests with different number of samples using the default parameters in Trinity v. 2.8.5^68^. A total of five individuals per species was chosen including the three regions of the brain (Supplementary Table S2a). To assess the quality of the resultant assemblies for each species, we investigated the read representation against our de novo assemblies using the aligner Bowtie2 v. 2.3.4.1^69^, with the*—very-sensitive* parameter. To reduce the transcript redundancy and to obtained only coding transcripts, we used the software transdecoder v. 5.5.0^68^ to identify the candidate coding regions ORF (open reading frame), keeping the option -*single_best_only*. From these results, we retrieved the output file with the final candidate ORF regions of more than 100 bp and then conducted a BLAST analysis using the Swissprot/Uniprot and Zebrafish databases obtained from www.uniprot.org (Swiss-Prot: November 2019) and NCBI (*Danio rerio*, txid7955, Apr 2018), respectively. Moreover, we explored the completeness of our assemblies using BUSCO v3^70^ to obtain the number of conserved ortholog content from our results represented in the dataset *Actinopterygiiodb9*. Finally, we computed the Nx statistics to estimate the approximate length of the transcripts in each assembly (N50) using the trinity script *trinity.stats.pl.* The N50 statistic provides information on the length of at least half (50%) of the total assembled transcripts. Consequently, the assembly for each species with the most complete and longest transcripts and with at least 95% of protein recovery was chosen as a reference (Supplementary Table S3).

We annotated the transcripts of the final de novo transcriptome assemblies for each species using BLAST + 2.10.0: December 16, 2019, and the databases Swissprot/Uniprot protein database (November 29, 2019), Zebrafish (*Danio rerio,* release Apr 2018) for both species, and the Ballan wrasse (*Labrus bergylta*, release March 2020) for *L. dimidiatus* only, obtained from Ensembl (GCA_900080235.1). We used *L. bergylta* because it is the closest species to *L. dimidiatus* with an available genome annotation. Finally, we used Omicsbox v. 1.3^71^ to functionally annotate the transcripts with Gene Ontology (GO terms) and KEGG pathways. Molecular pathways maps were not used directly from the KEGG software.

### Differential expression analyses

To perform differential expression analyses, we quantified transcript abundance for each species using the trinity script *align_and_estimate_abundance.pl* using RSEM v1.3.3 as quantification method and Bowtie2^69^ as mapping tool. Of the final gene expression matrix we filtered out transcripts with no expression by using the script *filter_low_expr_transcripts.pl* while retaining the most highly expressed isoforms for each gene using the command *–highest_iso_only*. To statistically evaluate differential gene expression, we used DESeq2-package v. 1.26.0^72^ with a Wald test statistic with a *design* =  ~ *treatment* for each region of the brain separately, an FDR p-adjusted significance value of 0.05 and an absolute log2fold change threshold of 0.3 as a cut-off as previously used in other study evaluating fish brain transcriptomics^26^ and to remove further potentially spurious significant differential expression and outliers. We compared the individuals from control vs interaction to determine their significant differential gene expression for the three regions of the brain Forebrain (FB), Midbrain (MB) and Hindbrain (HB) to retrieve the molecular response specific to these areas for both species, but separately for each species. Once statistically significant differentially expressed genes (DEGs) were obtained, functional enrichments were performed using Fisher’s exact test by testing the DEG subsets against the whole de novo transcriptome with a cut-off of FDR 0.05 in Omicsbox v. 1.3. Each fish species was analysed separately to capture the full breadth of differential gene expression per species due to the fact that the molecular reactions vary across organisms. The GO term IDs obtained were used as a reference to interpret the over-represented or under-represented molecular functions underlying the differentially expressed genes during the interaction, according to the annotations from the universal PANTHER (Protein Analysis Through Evolutionary Relationships) Classification System. All figures in the manuscript were produced by the authors using R version 4.0.5^73^.

### Ethical note

This work was conducted under the approval of Faculdade de Ciências da Universidade de Lisboa animal welfare body (ORBEA—Statement 01/2017) and Direção-Geral de Alimentação e Veterinária (DGAV—Permit 2018-05-23-010275) following the requirements imposed by the Directive 2010/63/EU of the European Parliament and of the Council of 22 September 2010 on the protection of animals used for scientific purposes. A Material Transfer Agreement of biological samples (fish brains) was signed between MARE and KAUST.

## Supplementary Information

Supplementary Information.

## Data Availability

Raw sequencing files and de novo transcriptome assemblies (*L. dimidiatus* and *A. leucosternon*) have been submitted under the NCBI Bioproject number PRJNA726349. Benefits from this research accrue from the sharing of our data and results on public databases as described above.
