# Supplementary material for: Neuro-molecular characterization of fish cleaning interactions
Source: Sci Rep. 2022 May 19;12:8468. doi: 10.1038/s41598-022-12363-6 (PMC9119974; doi:10.1038/s41598-022-12363-6)
Supplement: Supplementary file 1 — Supplementary Information. [file 41598_2022_12363_MOESM1_ESM.pdf]

Neuro-molecular characterization of fish cleaning interactions

Ramírez-Calero[1], S., Paula, J. R. [1,2], Otjacques, E. [2,3], Rosa, R. [2], Ravasi, T. [4,5], Schunter C. [1]*

[1]*The Swire Institute of Marine Science, School of Biological Sciences, The University of Hong Kong, Pokfulam Rd, Hong Kong SAR*

[2]*MARE – Marine and Environmental Sciences Centre, Laboratório Marítimo da Guia, Faculdade de Ciências da Universidade de Lisboa, Av. Nossa Senhora do Cabo, 9392750-374, Cascais, Portugal*

[3]*Pacific Biosciences Research Center, Kewalo Marine Laboratory, University of Hawai'i at Manoa, Honolulu, HI, USA*

[4]*Marine Climate Change Unit, Okinawa Institute of Science and Technology Graduate University, 1919–1 Tancha, Onna-son, Okinawa 904–0495, Japan*

[5]*Australian Research Council Centre of Excellence for Coral Reef Studies, James Cook University, Townsville, Queensland, 4811, Australia*

**Correspondence to: Celia Schunter*

Swire Institute of Marine Science,

Division for Ecology and Biodiversity,

School of Biological Sciences,

The University of Hong Kong, Pokfulam, Hong Kong SAR

(celiaschunter@gmail.com)

SUPPLEMENTARY MATERIAL

FIGURES:

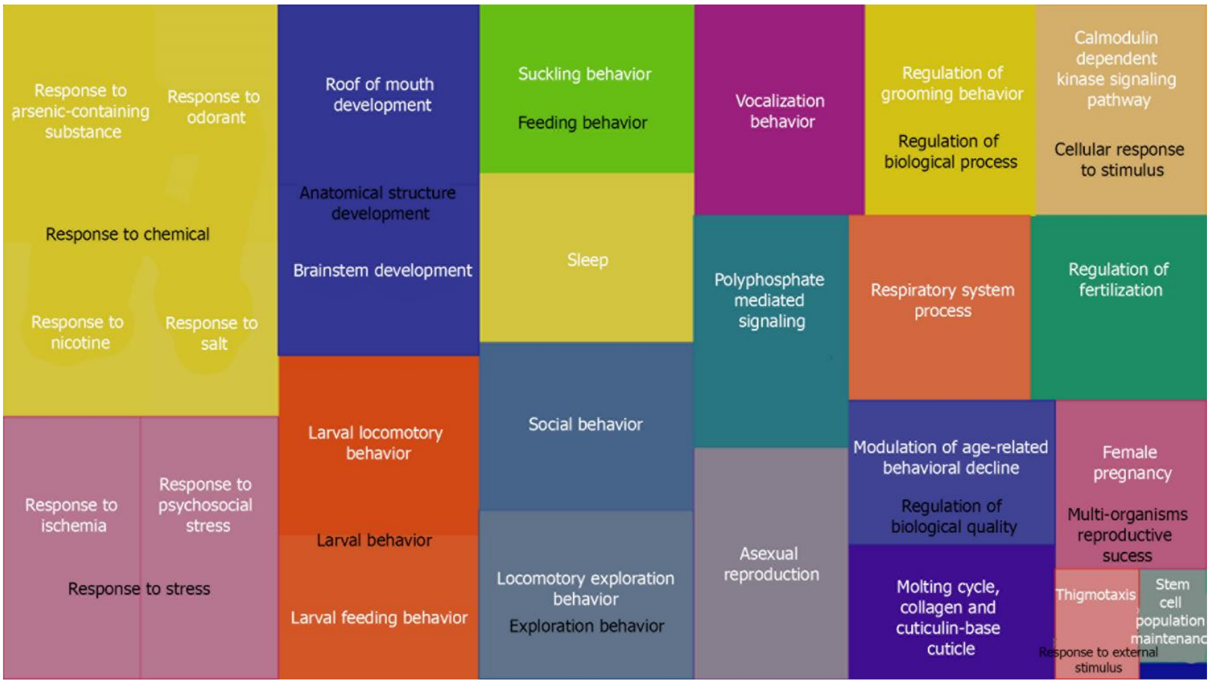

Figure S1. Gene Ontology treemap for *L. dimidiatus* representing the commonly significantly enriched functions in the forebrain region during the interaction treatment. Boxes with the same colour correspond to the upper-hierarchy GO-term and its title is found in the middle of each box.

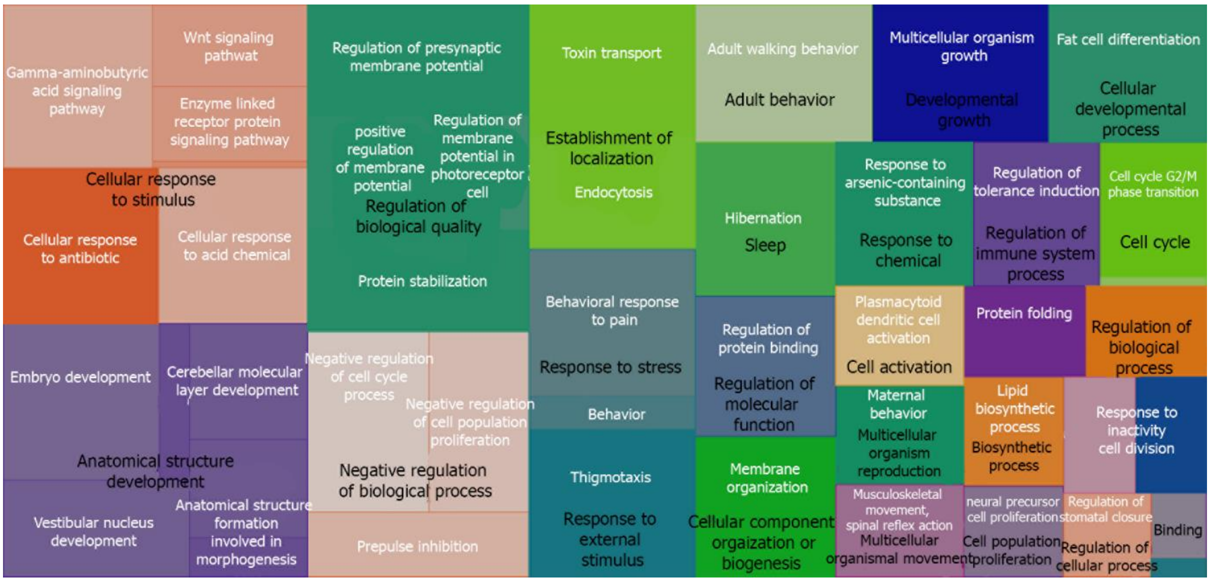

Figure S2. Gene Ontology treemap for *L. dimidiatus* representing the commonly significant enriched functions in the midbrain region during the interaction treatment. Boxes with the same colour correspond to the upper-hierarchy GO-term and its title is found in the middle of each box.

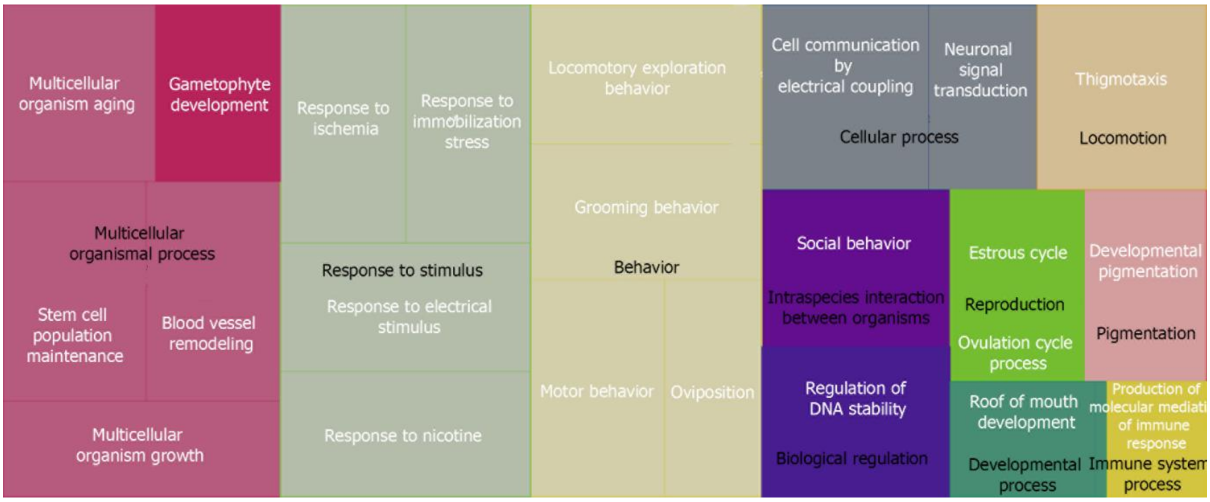

Figure S3. Gene Ontology treemap for *L. dimidiatus* representing the commonly significant enriched functions in the hindbrain region during the interaction treatment. Boxes with the same colour correspond to the upper-hierarchy GO-term and its title is found in the middle of each box.

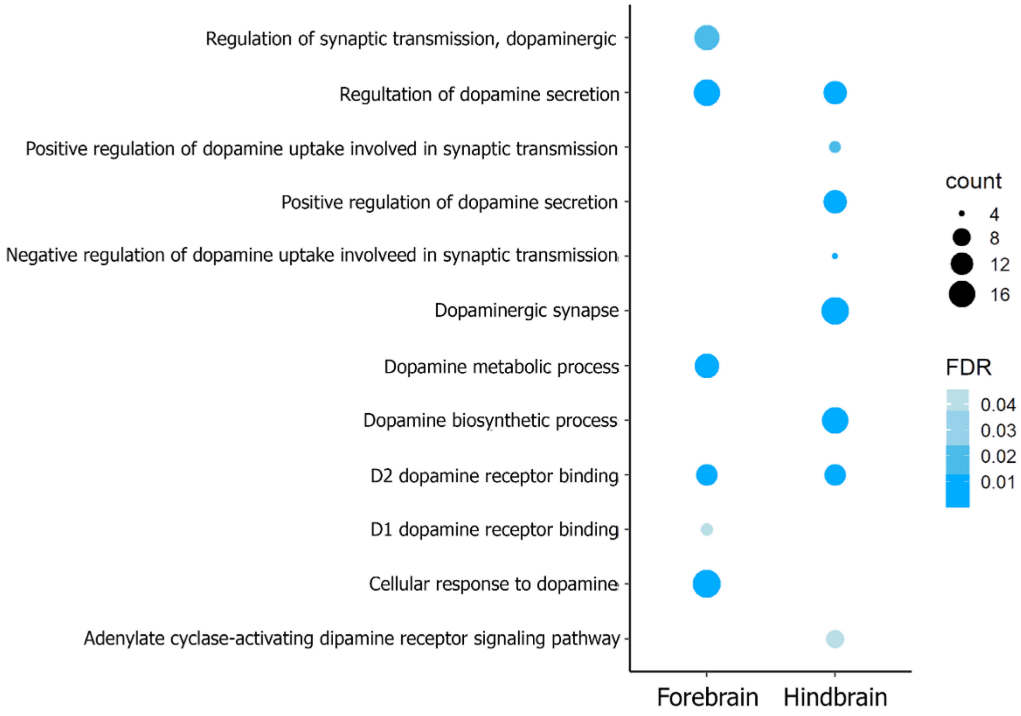

Figure S4. Functional enrichment of Gene Ontology (GO) terms related to Dopamine activity in *L. dimidiatus* in the fore and hindbrain. No enrichment was found for the midbrain region. The size of the circles is proportional to the number of genes observed within each GO category, and the colour of the circles is proportional to the significance (FDR value)

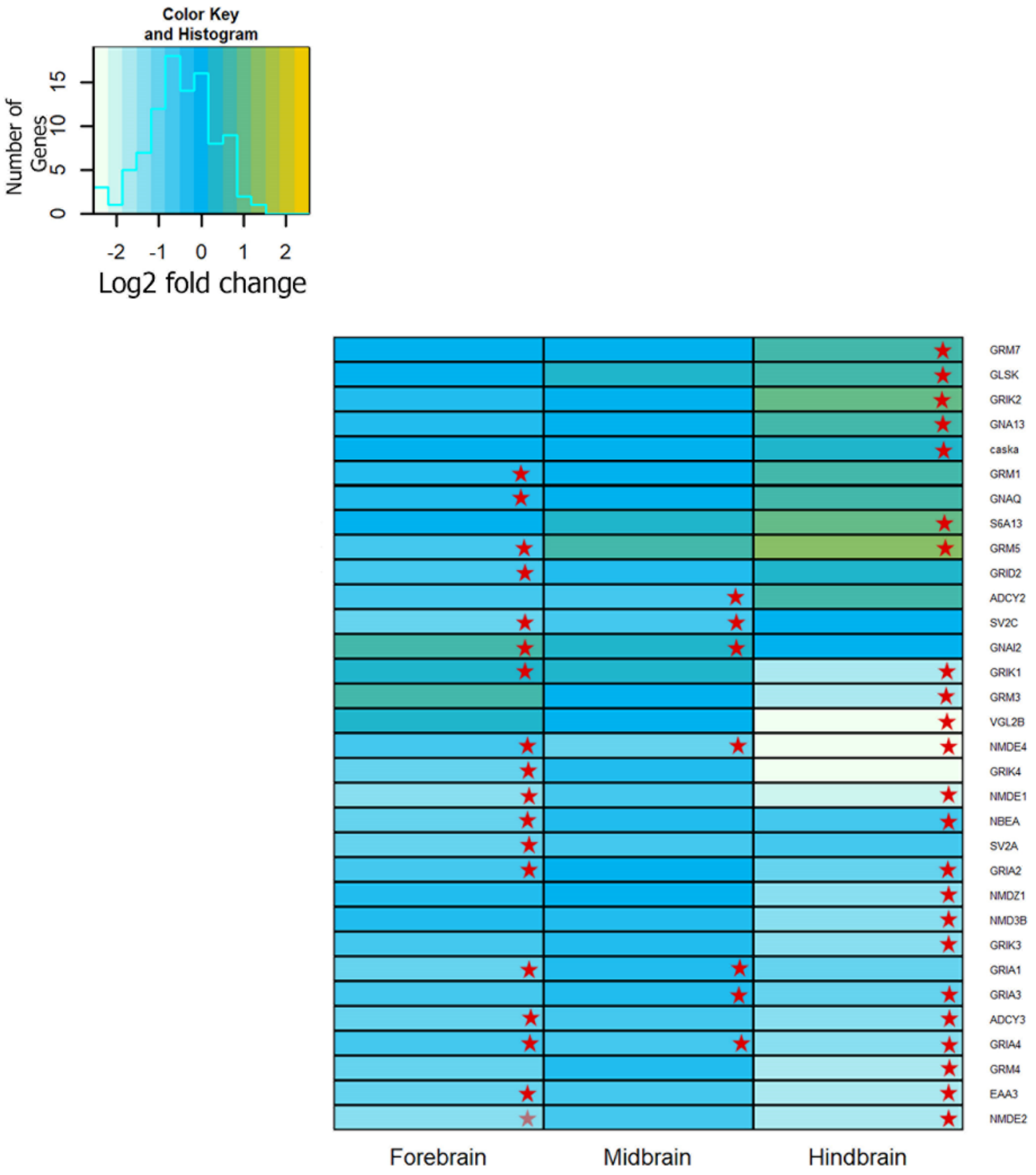

Figure S5. Comparative differential gene expression patterns of Glutamatergic synapse genes in the regions of the brain of *L. dimidiatus*. Red stars represent significance (padj<0.05), while colours represent log2fold change estimates (threshold of 0.3). Gene ADCY2 (Adenylate cyclase type 2), ADCY3 (Adenylate cyclase type 3), CA2D1 (Voltage-dependent calcium channel subunit alpha-2/delta-1), CA2D2 (Voltage-dependent calcium channel subunit alpha-2/delta-2), CABP7 (Calcium-binding protein 7), CAC1A (Voltage-dependent P/Q-type calcium channel subunit alpha-1A), CAC1B (Probable voltage-dependent N-type calcium channel subunit alpha-1B), CAC1C (Voltage-dependent L-type calcium channel subunit alpha-1C), CAC1D (Voltage-dependent L-type calcium channel subunit alpha-1D), CAC1G (Voltage-dependent T-type calcium channel subunit alpha-1G), CAC1H (Voltage-dependent T-type calcium channel subunit alpha-1H), CAC1I (Voltage-dependent T-type calcium channel subunit alpha-1I), CACB2 (Voltage-dependent L-type calcium channel subunit beta-2), CASKA (calcium/calmodulin dependent serine protein kinase), CCG2 (Voltage-dependent calcium channel gamma-2 subunit), CCG4 (Voltage-dependent calcium channel gamma-4 subunit), CCG5 (Voltage-dependent calcium channel gamma-5 subunit), CCG8 (Voltage-

dependent calcium channel gamma-8 subunit) CPLX2 (Calcium/calmodulin-dependent protein kinase type 1), CSKP (Calcium/calmodulin-dependent protein kinase type 1D), EAA3 (Excitatory amino acid transporter 3), GABR1 (Gamma-aminobutyric acid type B receptor subunit 1), GABR2 (Gamma-aminobutyric acid type B receptor subunit 2), GBLP (Guanine nucleotide-binding protein subunit beta-2-like 1), GBRB4 (Gamma-aminobutyric acid receptor subunit beta-4), GBRG1 (Gamma-aminobutyric acid receptor subunit gamma-1), GBRP (Gamma-aminobutyric acid receptor subunit pi), GBRR1 (Gamma-aminobutyric acid receptor subunit rho-1), GBRR2 (Gamma-aminobutyric acid receptor subunit rho-2), GCR (Glucocorticoid receptor), GCYA1 (Guanylate cyclase soluble subunit alpha-1), GCYA2 (Guanylate cyclase soluble subunit alpha-2), GCYB1 (Guanylate cyclase soluble subunit beta-1), GLRA1 (Glycine receptor subunit alphaZ1), GLRA2 (Glycine receptor subunit alpha-2), GLRA4 (Glycine receptor subunit alpha-4), GLRB (Glycine receptor subunit beta), GLSK (Glutaminase kidney isoform, mitochondrial), GNA13 (Guanine nucleotide-binding protein subunit alpha-13), GNAI1 (Guanine nucleotide-binding protein G(i) subunit alpha-1), GNAI2 (Guanine nucleotide-binding protein G(i) subunit alpha-2), GNAQ (BELL-associated factor 1), GNB5A (Guanine nucleotide-binding protein subunit beta-5a), GNL1 (Guanine nucleotide-binding protein-like 1), GRIA1 (Glutamate receptor 1), GRIA2 (Glutamate receptor 2), GRIA3 (Glutamate receptor 3), GRIA4 (Glutamate receptor 4), GRID2 (Glutamate receptor ionotropic, delta-2), GRIK1 (Glutamate receptor ionotropic, kainate 1, GRIK2 (Glutamate receptor ionotropic, kainate 2), GRIK3 (Glutamate receptor ionotropic, kainate 3), GRIK4 (Glutamate receptor ionotropic, kainate 4), GRM1 (Metabotropic glutamate receptor 1), GRM3 (Metabotropic glutamate receptor 3), GRM4 (Metabotropic glutamate receptor 4), GRM5 (Metabotropic glutamate receptor 5, GRM7 (Metabotropic glutamate receptor 7, KAPCA (cAMP-dependent protein kinase catalytic subunit alpha), KC2D2 (Calcium/calmodulin-dependent protein kinase type II delta 2 chain), KCC1D (Calcium/calmodulin-dependent protein kinase type 1D), KCC1G (Calcium/calmodulin-dependent protein kinase type 1G), KCC2A (Gamma-aminobutyric acid receptor subunit beta-3), NAC1 (Voltage-dependent P/Q-type calcium channel subunit alpha-1A), NBEA (Glucocorticoid receptor), NMD3B (Glutamate receptor ionotropic, NMDA 3B), NMDE1 (Glutamate receptor ionotropic, NMDA 2A), NMDE2 (Glutamate receptor ionotropic, NMDA 2B), NMDE4 (Glutamate receptor ionotropic, NMDA 2D), NMDZ1 (Glutamate receptor ionotropic, NMDA 1), S6A13 (Sodium- and chloride-dependent GABA transporter 2), SV2A (Synaptic vesicle glycoprotein 2A), SV2C (Synaptic vesicle glycoprotein 2C), VGL2B (Vesicular glutamate transporter 2.2). The legend indicates the reference values of log2fold changes for each DEG in the figure.

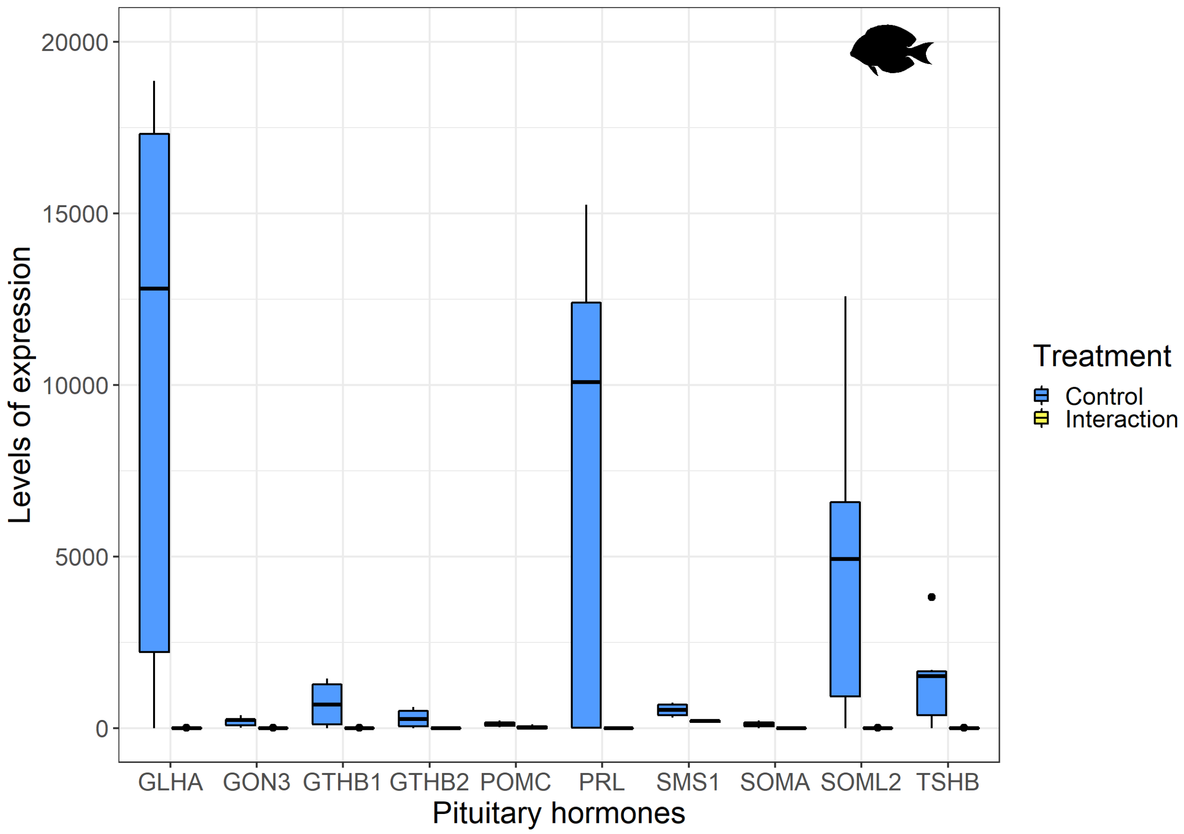

Figure S6. Gene expression levels of significant Hypothalamic-Pituitary-Thyroid (HPT) hormone genes in the forebrain region of *Acanthurus leucosternon* during the interaction with *L. dimidiatus*. Gene GLHA (Glycoprotein hormones alpha chain), GON3 (Progonadoliberin-3), GTHB1 (Gonadotropin subunit beta-1), GTHB2 (Gonadotropin subunit beta-2), POMC (Pro-opiomelanocortin), PRL (Prolactin), SMS1 (Somatostatin-1), SOMA (Somatotropin), SOML2 (Somatolactin-2), TSHB (Thyrotropin subunit beta).

TABLES

Table S1a. Information of individuals of *L. dimidiatus* and *A. leucosternon* that were used for each experimental setup: Control and Interaction.

ID	Date	Hour of dissection (am)	Species	Experimental setup	Interaction partner	Standard length (cm)	Weight (g)
AL31	7 Aug	8:45	*Acanthurus leucosternon*	Interaction	LD9	10.6	49.15
AL32	7 Aug	8:45	*Acanthurus leucosternon*	Interaction	LD10	9.2	33.47
AL33	7 Aug	8:45	*Acanthurus leucosternon*	Interaction	LD7	25.46	11.4
AL34	7 Aug	8:45	*Acanthurus leucosternon*	Interaction	LD8	14.73	9.7
AL35	7 Aug	9:30	*Acanthurus leucosternon*	Interaction	LD1	10.3	47.6
AL36	7 Aug	9:30	*Acanthurus leucosternon*	Interaction	LD2	13.7	57.36
AL37	7 Aug	9:30	*Acanthurus leucosternon*	Interaction	LD4	7.4	16.98
AL38	7 Aug	9:30	*Acanthurus leucosternon*	Interaction	LD3	7.5	17.06
AL39	7 Aug	10:15	*Acanthurus leucosternon*	Control	NA	7.2	16.02
AL40	7 Aug	10:15	*Acanthurus leucosternon*	Control	NA	8.6	27.23
AL41	7 Aug	10:15	*Acanthurus leucosternon*	Interaction	LD19	10.5	51.32
AL42	7 Aug	10:15	*Acanthurus leucosternon*	Interaction	LD20	9.2	33.23
AL43	8 Aug	8:45	*Acanthurus leucosternon*	Interaction	LD17	28	11.7
AL44	8 Aug	8:45	*Acanthurus leucosternon*	Interaction	LD18	22.6	11.1
AL45	8 Aug	8:45	*Acanthurus leucosternon*	Interaction	LD11	10.7	58.19
AL46	8 Aug	8:45	*Acanthurus leucosternon*	Interaction	LD12	12.6	33.95
AL47	8 Aug	9:30	*Acanthurus leucosternon*	Interaction	LD4	7.2	15.96
AL48	8 Aug	9:30	*Acanthurus leucosternon*	Control	NA	7	13.62
AL49	8 Aug	9:30	*Acanthurus leucosternon*	Control	NA	7	13.94
AL50	8 Aug	9:30	*Acanthurus leucosternon*	Interaction	LD3	10.5	52.24
AL51	8 Aug	10:15	*Acanthurus leucosternon*	Interaction	LD29	9.3	32.03
AL52	8 Aug	10:15	*Acanthurus leucosternon*	Interaction	LD30	9.4	37.59
AL53	8 Aug	10:15	*Acanthurus leucosternon*	Interaction	LD27	24.02	11
AL54	8 Aug	10:15	*Acanthurus leucosternon*	Interaction	LD28	22.95	11.1
AL55	9 Aug	8:45	*Acanthurus leucosternon*	Interaction	LD21	13.9	52.87
AL56	9 Aug	8:45	*Acanthurus leucosternon*	Interaction	LD22	13.4	47.29
AL57	9 Aug	8:45	*Acanthurus leucosternon*	Interaction	LD4	7.2	17.15
AL58	9 Aug	8:45	*Acanthurus leucosternon*	Control	NA	8.2	24.31
AL59	9 Aug	9:30	*Acanthurus leucosternon*	Control	NA	6.9	14.36
AL60	9 Aug	9:30	*Acanthurus leucosternon*	Interaction	LD3	10.5	56.52
LD10	9 Aug	9:30	*Labroides dimidiatus*	Interaction	AL32	6	2.4
LD11	9 Aug	9:30	*Labroides dimidiatus*	Interaction	AL45	6	2.26
LD12	9 Aug	10:15	*Labroides dimidiatus*	Interaction	AL46	7.93	2.3
LD13	9 Aug	10:15	*Labroides dimidiatus*	Interaction	AL20	6.3	2.77
LD14	9 Aug	10:15	*Labroides dimidiatus*	Interaction	AL17	5.4	1.92
LD15	9 Aug	10:15	*Labroides dimidiatus*	Control	NA	6.8	2.74
LD16	10 Aug	8:45	*Labroides dimidiatus*	Control	NA	6	2.19
LD17	10 Aug	8:45	*Labroides dimidiatus*	Interaction	AL43	1.91	6.6
LD18	10 Aug	9:30	*Labroides dimidiatus*	Interaction	AL44	1.49	5.8
LD19	10 Aug	9:30	*Labroides dimidiatus*	Interaction	AL41	6	2.26
LD1	10 Aug	10:15	*Labroides dimidiatus*	Interaction	AL35	6	2.61

LD20	10 Aug	10:15	*Labroides dimidiatus*	Interaction	AL42	5.5	1.84
LD21	11 Aug	8:45	*Labroides dimidiatus*	Interaction	AL55	7.7	2.81
LD22	11 Aug	8:45	*Labroides dimidiatus*	Interaction	AL56	7.2	2.17
LD23	11 Aug	9:30	*Labroides dimidiatus*	Interaction	AL20	6	2.3
LD24	11 Aug	9:30	*Labroides dimidiatus*	Interaction	AL17	6.1	2.82
LD25	11 Aug	10:15	*Labroides dimidiatus*	Control	NA	6	2.73
LD26	11 Aug	10:15	*Labroides dimidiatus*	Control	NA	6	2.59
LD27	12 Aug	8:45	*Labroides dimidiatus*	Interaction	AL53	1.61	6.8
LD28	12 Aug	8:45	*Labroides dimidiatus*	Interaction	AL54	1.71	6.3
LD29	12 Aug	8:45	*Labroides dimidiatus*	Interaction	AL51	6	2.59
LD2	12 Aug	8:45	*Labroides dimidiatus*	Interaction	AL36	6.8	1.9
LD30	12 Aug	9:30	*Labroides dimidiatus*	Interaction	AL52	5.2	1.46
LD3	12 Aug	9:30	*Labroides dimidiatus*	Interaction	AL18	5.7	1.92
LD4	12 Aug	9:30	*Labroides dimidiatus*	Interaction	AL17	5.1	1.57
LD5	13 Aug	9:30	*Labroides dimidiatus*	Control	NA	6.2	2.98
LD6	14 Aug	10:15	*Labroides dimidiatus*	Control	NA	6	2.21
LD7	15 Aug	10:15	*Labroides dimidiatus*	Interaction	AL33	2.18	7
LD8	16 Aug	10:15	*Labroides dimidiatus*	Interaction	AL34	27.1	7.3
LD9	17 Aug	10:15	*Labroides dimidiatus*	Interaction	AL31	5.7	1.89

Table S1b. Ethogram with behavioural codes and definitions used for behavioural analysis with BORIS.

Behaviour type	Shortcut key	Behaviour code	Description	Behavioural Category
State event	3	TS	Tactile Stimulation	Interaction Quality
State event	1	Int_st_cleaners	Interactions started by cleaners	Motivation
State event	2	Int_st_clients	Interactions started by clients	Motivation
Point event	4	Pousing	Client Pousing	Motivation
Point event	5	Jolt	Jolt	Interaction Quality
Point event	6	Chase	Chase	Interaction Quality

Table S1c. Behavioural trial data for each aquarium setup for individuals of *L. dimidiatus* and *A. leucosternon* assigned for control (no interaction) and interaction treatment. NCBI Biosample accession numbers and video of behavioural trials are provided.

ID	Video link	Biosample Accession number *Labroides dimidiatus* (fore-, mid- and hindbrain)	Biosample Accession number *Acanthurus leucosternon* (fore-, mid- and hindbrain)	Experiment Setup	Video duration (min)	N. interactions	N. interactions started by the cleaner	Proportion of interactions started by the cleaner (%)	Number of posing	Total duration of the interaction (sec)	Mean duration of interaction (sec)	Proportion of time spent interacting (%)	Number of jolts	Number of chase	N. tactile stimulation	Tactile stimulation (sec)
AL39	https://youtu.be/3JImyTU5qYM		SRR14415113 SRR14415112 SRR14415111	Control	36.0	0	0	0	0	0	0	0	0	0	0	0
AL40	https://youtu.be/q4rOhyXFh3c		SRR14415110 SRR14415109 SRR14415108	Control	40.2	0	0	0	0	0	0	0	0	0	0	0
AL48	https://youtu.be/CCj_HCek_zk		SRR14415083 SRR14415082 SRR14415081	Control	44.6	0	0	0	0	0	0	0	0	0	0	0
AL49	https://youtu.be/lsoSeHKeg7c		SRR14415080 SRR14415079 SRR14415078	Control	40.8	0	0	0	0	0	0	0	0	0	0	0
AL58	https://youtu.be/cngmTc2MoSA		SRR14415050 SRR14415049 SRR14415048	Control	46.2	0	0	0	0	0	0	0	0	0	0	0
AL59	https://youtu.be/ODEnSJ8k3ts		SRR14415047 SRR14415046 SRR14415045	Control	36.5	0	0	0	0	0	0	0	0	0	0	0
LD15	https://youtu.be/CypMkVVLMdk	SRR14415191 SRR14415190 SRR14415189		Control	49.4	0	0	0	0	0	0	0	0	0	0	0
LD16	https://youtu.be/i02KGsFJmhQ	SRR14415188 SRR14415187 SRR14415186		Control	47.7	0	0	0	0	0	0	0	0	0	0	0

LD25	https://youtu.<br>be/6-<br>fPvCsugu0	SRR14415158<br>SRR14415157<br>SRR14415156		Control	48.8	0	0	0	0	0	0	0	0	0	0	0
LD26	https://youtu.<br>be/N70YAp8<br>NmnA	SRR14415155<br>SRR14415154<br>SRR14415153		Control	44.6	0	0	0	0	0	0	0	0	0	0	0
LD5	https://youtu.<br>be/tnFUINbu<br>p4I	SRR14415107<br>SRR14415096<br>SRR14415085		Control	42.9	0	0	0	0	0	0	0	0	0	0	0
LD6	https://youtu.<br>be/i02KGsFJ<br>mhQ	SRR14415074<br>SRR14415063<br>SRR14415052		Control	18.0	0	0	0	0	0	0	0	0	0	0	0
LD03<br>AL38	https://youtu.<br>be/nb6C-<br>Gks8_M	SRR14415172<br>SRR14415161<br>SRR14415150	SRR14415116<br>SRR14415115<br>SRR14415114	Interaction	41.3	69	40	58	55	456	7	18.4	1	1	0	0
LD04<br>AL37	https://youtu.<br>be/VJot-<br>M0khMI	SRR14415139<br>SRR14415210<br>SRR14415118	SRR14415120<br>SRR14415119<br>SRR14415117	Interaction	49.5	63	37	59	46	531	8	17.9	14	10	13	27
LD13<br>AL50	https://youtu.<br>be/vsb0jtUF<br>YyI	SRR14415198<br>SRR14415197<br>SRR14415196	SRR14415077<br>SRR14415076<br>SRR14415075	Interaction	54.8	81	76	94	8	690	9	21.0	4	1	2	5
LD14<br>AL47	https://youtu.<br>be/x2vqfbI2L<br>Hg	SRR14415192<br>SRR14415195<br>SRR14415193	SRR14415087<br>SRR14415086<br>SRR14415084	Interaction	38.2	34	28	82	9	180	5	7.9	0	0	0	0
LD23<br>AL60	https://youtu.<br>be/bdATUG9<br>6w9Y	SRR14415165<br>SRR14415164<br>SRR14415163	SRR14415044<br>SRR14415043<br>SRR14415042	Interaction	40.3	30	15	50	17	267	9	11.1	2	1	8	40
LD24<br>AL57	https://youtu.<br>be/hnF_26W<br>nqHg	SRR14415162<br>SRR14415160<br>SRR14415159	SRR14415054<br>SRR14415053<br>SRR14415051	Interaction	48.2	50	44	88	8	324	6	11.2	2	2	1	1

Table S2a. Number of reads of sample individuals of *L. dimidiatus* and *A. leucosternon* used for the De novo transcriptome assembly before and after removing illumina adapters with Trimmomatic v.0.36.

Species	ID	Before quality clean up (R1/R2)	After quality clean up(R1/R2)
Labroides dimidiatus	LD10FB	35280159	35255745
Labroides dimidiatus	LD10MB	38914076	38881875
Labroides dimidiatus	LD10HB	29704418	29685972
Labroides dimidiatus	LD17FB	44595130	44573048
Labroides dimidiatus	LD17MB	32001272	31559451
Labroides dimidiatus	LD17HB	29968877	29929743
Labroides dimidiatus	LD11FB	37710260	37684137
Labroides dimidiatus	LD11MB	27734874	27697716
Labroides dimidiatus	LD11HB	23911684	23891801
Labroides dimidiatus	LD13FB	33691852	33665951
Labroides dimidiatus	LD13MB	28233929	28203709
Labroides dimidiatus	LD13HB	26963793	26921849
Labroides dimidiatus	LD15FB	27758342	27736193
Labroides dimidiatus	LD15MB	30974352	30951472
Labroides dimidiatus	LD15HB	24473282	24446693
Acanthurus leucosternon	AL51FB	39515162	39492072
Acanthurus leucosternon	AL51MB	37935891	37843553
Acanthurus leucosternon	AL51HB	30949210	30871702
Acanthurus leucosternon	AL53FB	36135759	36109008
Acanthurus leucosternon	AL53MB	39316484	39273057
Acanthurus leucosternon	AL53HB	34301181	34277364
Acanthurus leucosternon	AL46FB	33532746	33432754
Acanthurus leucosternon	AL46MB	30510385	30488045
Acanthurus leucosternon	AL46HB	25962178	25940874
Acanthurus leucosternon	AL49FB	33503475	33459768
Acanthurus leucosternon	AL49MB	29668287	29649314
Acanthurus leucosternon	AL49HB	38033891	37527978
Acanthurus leucosternon	AL50FB	31861643	31840621
Acanthurus leucosternon	AL50MB	31791966	31763546
Acanthurus leucosternon	AL50HB	35926528	35877444

Table S2b. *De novo* transcriptome assembly metrics for *L. dimidiatus* and *A. leucosternon*

Metrics	Samples used	Average number of assembled reads	Overall alignment rate (%)	Total trinity genes	Transcripts number before transdecoder	Total assembled bases	Average contig	Median contig length	N50
L. dimidiatus	5	31405690.33	95.49	356122	594678	549131976	923.41	441	1813
A. leucosternon	5	33856473.33	95.15	349774	513852	623652961	1213.68	501	2827

Table S3. Quality metrics for *de novo* assembly of the transcriptome for *L. dimidiatus* and *A. leucosternon*.

Quality Assessment	Transcripts number before transdecoder	Overall alignment rate (%)	Transcript number after transdecoder	Swissprot/Uniprot	D. rerio	L. bergylta	Complete BUSCOs	Complete and single-copy BUSCOs	Complete and duplicated BUSCOs
L. dimidiatus	594678	95.49	114687	26380	8379	28988	4260 (92.9%)	1372 (29.9%)	2888 (63%)
A. leucosternon	513852	95.15	123839	27372	6438	30770	4316 (94%)	1599 (34.8%)	2717 (59.2%)

Table S4. Top 100 significant enriched functions based on the differentially expressed genes shared across the three brain regions in *L. dimidiatus* during the interaction treatment

GO ID	GO Name	GO Category	FDR	P-Value	Nr Test	Nr Reference
GO:0032839	Dendrite cytoplasm	CELLULAR	0.006114001	1.76E-06	5	177
GO:0003729	MRna binding	MOLECULAR	0.013925266	1.29E-05	7	712
GO:0015081	Sodium ion transmembrane transporter activity	MOLECULAR	0.014511955	1.41E-05	6	474
GO:0005524	ATP binding	MOLECULAR	0.014897318	1.95E-05	11	2181
GO:0061003	Positive regulation of dendritic spine morphogenesis	BIOLOGICAL	0.014897318	2.02E-05	4	141
GO:0043025	Neuronal cell body	CELLULAR	0.016375155	2.48E-05	13	3152
GO:0042802	Identical protein binding	MOLECULAR	0.016423969	2.61E-05	18	5892
GO:0010038	Response to metal ion	BIOLOGICAL	0.021606521	4.50E-05	10	1958
GO:0055037	Recycling endosome	CELLULAR	0.021606521	4.39E-05	6	582
GO:0098989	NMDA selective glutamate receptor signalling pathway	BIOLOGICAL	0.0224743	4.96E-05	3	61
GO:1902306	Negative regulation of sodium ion transmembrane transport	BIOLOGICAL	0.023137697	5.19E-05	3	62
GO:0005654	Nucleoplasm	CELLULAR	0.025920389	6.72E-05	20	7605
GO:0048878	Chemical homeostasis	BIOLOGICAL	0.025920389	6.36E-05	14	3974
GO:0040020	Regulation of meiotic nuclear division	BIOLOGICAL	0.025920389	6.69E-05	4	193
GO:0099522	Region of cytosol	CELLULAR	0.025920389	6.56E-05	4	192
GO:0060255	Regulation of macromolecule metabolic process	BIOLOGICAL	0.027453147	7.61E-05	26	12003
GO:0031323	Regulation of cellular metabolic process	BIOLOGICAL	0.027453147	7.80E-05	26	12019
GO:0015079	Potassium ion transmembrane transporter activity	MOLECULAR	0.027453147	7.86E-05	5	396
GO:1990446	U1 snrnp binding	MOLECULAR	0.033305905	1.07E-04	2	12
GO:0030619	U1 snrna binding	MOLECULAR	0.033305905	1.07E-04	2	12
GO:0045211	Postsynaptic membrane	CELLULAR	0.034986381	1.16E-04	8	1369
GO:1902514	Regulation of calcium ion transmembrane transport via high voltage-gated calcium channel	BIOLOGICAL	0.034986381	1.16E-04	3	82

GO:0051259	Protein complex oligomerization	BIOLOGICAL	0.035630156	1.25E-04	6	706
GO:1905375	Cellular response to homocysteine	BIOLOGICAL	0.035630156	1.23E-04	2	13
GO:0098978	Glutamatergic synapse	CELLULAR	0.036631692	1.33E-04	10	2232
GO:0043169	Cation binding	MOLECULAR	0.037843578	1.44E-04	16	5449
GO:0010008	Endosome membrane	CELLULAR	0.037843578	1.43E-04	7	1045
GO:1990443	Peptidyl-threonine autophosphorylation	BIOLOGICAL	0.037843578	1.41E-04	2	14
GO:0051649	Establishment of localization in cell	BIOLOGICAL	0.037879499	1.50E-04	18	6713
GO:0019228	Neuronal action potential	BIOLOGICAL	0.03825319	1.52E-04	4	240
GO:0110088	Hippocampal neuron apoptotic process	BIOLOGICAL	0.03961376	1.59E-04	2	15
GO:0008104	Protein localization	BIOLOGICAL	0.041907049	1.88E-04	19	7491
GO:0071702	Organic substance transport	BIOLOGICAL	0.041907049	1.88E-04	18	6832
GO:0044703	Multi-organism reproductive process	BIOLOGICAL	0.041907049	1.74E-04	14	4365
GO:0099536	Synaptic signalling	BIOLOGICAL	0.041907049	1.88E-04	12	3310
GO:0098889	Intrinsic component of presynaptic membrane	CELLULAR	0.041907049	1.75E-04	6	751
GO:0034706	Sodium channel complex	CELLULAR	0.041907049	1.82E-04	3	96
GO:2000124	Regulation of endocannabinoid signalling pathway	BIOLOGICAL	0.041907049	1.79E-04	2	16
GO:0071705	Nitrogen compound transport	BIOLOGICAL	0.04210399	1.92E-04	17	6204
GO:0003727	Single-stranded RNA binding	MOLECULAR	0.04631629	2.15E-04	4	263
GO:0005769	Early endosome	CELLULAR	0.048964054	2.29E-04	7	1130
GO:0048513	Animal organ development	BIOLOGICAL	0.049175849	2.45E-04	24	11238
GO:0065009	Regulation of molecular function	BIOLOGICAL	0.049175849	2.36E-04	19	7619
GO:0033044	Regulation of chromosome organization	BIOLOGICAL	0.049175849	2.39E-04	7	1138
GO:0002030	Inhibitory G protein-coupled receptor phosphorylation	BIOLOGICAL	0.049175849	2.46E-04	2	19

Table S5. Top 100 enriched GO terms for the Forebrain region (FB) of *L. dimidiatus* during the interaction treatment.

GO ID	GO Name	GO Category	FDR	P-Value	Nr Test	Nr Reference
GO:0098978	Glutamatergic synapse	CELLULAR	1.91E-104	5.10E-107	292	1950
GO:0098805	Membrane	CELLULAR	2.93E-62	2.02E-64	341	4055
GO:0098693	Regulation of synaptic vesicle cycle	BIOLOGICAL	6.08E-41	7.90E-43	112	686
GO:0098685	Schaffer collateral - CA1 synapse	CELLULAR	2.84E-36	4.34E-38	97	577
GO:0031594	Neuromuscular junction	CELLULAR	4.55E-36	7.00E-38	95	555
GO:0005524	ATP binding	MOLECULAR	4.80E-35	7.63E-37	193	2207
GO:0045666	Positive regulation of neuron differentiation	BIOLOGICAL	8.20E-35	1.31E-36	183	2024
GO:0031267	Small gtpase binding	MOLECULAR	2.12E-29	3.92E-31	117	1033
GO:0005516	Calmodulin binding	MOLECULAR	6.61E-29	1.26E-30	94	688
GO:0023014	Signal transduction	BIOLOGICAL	9.58E-28	1.90E-29	197	2623
GO:0030165	PDZ domain binding	MOLECULAR	3.79E-27	7.70E-29	78	501
GO:0098982	GABA-ergic synapse	CELLULAR	1.42E-26	3.01E-28	73	446
GO:0099092	Postsynaptic density, intracellular component	CELLULAR	3.45E-26	7.41E-28	43	125
GO:0043524	Negative regulation of neuron apoptotic process	BIOLOGICAL	1.63E-25	3.62E-27	83	607
GO:0043204	Perikaryon	CELLULAR	1.93E-25	4.31E-27	93	760
GO:0098688	Parallel fiber to Purkinje cell synapse	CELLULAR	2.55E-25	5.74E-27	51	208
GO:0008022	Protein C-terminus binding	MOLECULAR	4.48E-25	1.02E-26	99	866
GO:0017016	Small gtpase binding	MOLECULAR	2.52E-24	5.92E-26	104	972
GO:0099061	Integral component of postsynaptic density membrane	CELLULAR	5.20E-24	1.23E-25	61	340
GO:0043198	Dendritic shaft	CELLULAR	1.71E-23	4.13E-25	61	349
GO:1900006	Positive regulation of dendrite development	BIOLOGICAL	4.18E-23	1.03E-24	69	462
GO:0042803	Protein homodimerization activity	MOLECULAR	2.09E-22	5.33E-24	150	1915
GO:0097110	Scaffold protein binding	MOLECULAR	3.69E-22	9.51E-24	65	427

GO:0047485	Protein N-terminus binding	MOLECULAR	1.35E-20	3.75E-22	65	461
GO:0098589	Membrane	CELLULAR	5.82E-20	1.67E-21	118	1391
GO:0044325	Ion channel binding	MOLECULAR	9.89E-20	2.87E-21	70	556
GO:0050885	Neuromuscular process controlling balance	BIOLOGICAL	1.82E-19	5.34E-21	54	334
GO:0001662	Behavioral fear response	BIOLOGICAL	2.37E-18	7.37E-20	44	229
GO:0016324	Apical plasma membrane	CELLULAR	3.21E-18	1.01E-19	108	1274
GO:0045665	Negative regulation of neuron differentiation	BIOLOGICAL	4.64E-18	1.47E-19	104	1204
GO:0010771	Negative regulation of cell morphogenesis involved in differentiation	BIOLOGICAL	4.69E-18	1.48E-19	62	478
GO:2000310	Regulation of NMDA receptor activity	BIOLOGICAL	1.71E-17	5.54E-19	40	196
GO:0098686	Hippocampal mossy fiber to CA3 synapse	CELLULAR	2.36E-16	8.21E-18	43	251
GO:0001540	Amyloid-beta binding	MOLECULAR	3.51E-16	1.23E-17	44	267
GO:0072358	Circulatory system development	BIOLOGICAL	6.54E-16	2.34E-17	168	2696
GO:0090630	Activation of gtpase activity	BIOLOGICAL	1.34E-15	4.89E-17	40	227
GO:0005509	Calcium ion binding	MOLECULAR	1.83E-15	6.75E-17	106	1365
GO:0016328	Lateral plasma membrane	CELLULAR	8.89E-15	3.41E-16	48	352
GO:0035176	Social behavior	BIOLOGICAL	1.26E-14	4.87E-16	37	207
GO:1904646	Cellular response to amyloid-beta	BIOLOGICAL	1.29E-14	5.00E-16	36	195
GO:0007605	Sensory perception of sound	BIOLOGICAL	2.71E-14	1.07E-15	75	816
GO:0017137	Small gtpase binding	MOLECULAR	1.25E-13	5.13E-15	49	396
GO:0008542	Visual learning	BIOLOGICAL	4.82E-13	2.05E-14	41	292
GO:1903078	Positive regulation of protein localization to plasma membrane	BIOLOGICAL	6.00E-13	2.56E-14	40	280
GO:0007616	Long-term memory	BIOLOGICAL	8.71E-13	3.76E-14	41	298
GO:0048013	Ephrin receptor signaling pathway	BIOLOGICAL	1.87E-12	8.25E-14	38	263
GO:0005730	Nucleolus	CELLULAR	3.64E-12	1.65E-13	136	2229
GO:0031901	Early endosome membrane	CELLULAR	6.15E-12	2.83E-13	41	319
GO:0051117	Atpase binding	MOLECULAR	2.56E-11	1.23E-12	42	351
GO:0005546	Phosphatidylinositol-4,5-bisphosphate binding	MOLECULAR	3.54E-11	1.72E-12	42	355

GO:0005813	Centrosome	CELLULAR	3.67E-11	1.78E-12	96	1396
GO:0046982	Protein heterodimerization activity	MOLECULAR	4.15E-11	2.02E-12	67	801
GO:0017124	SH3 domain binding	MOLECULAR	5.62E-11	2.77E-12	41	345
GO:0005085	Guanyl-nucleotide exchange factor activity	MOLECULAR	6.50E-11	3.21E-12	46	428
GO:0035641	Locomotory exploration behavior	BIOLOGICAL	2.34E-10	1.20E-11	20	79
GO:0071625	Vocalization behavior	BIOLOGICAL	3.82E-10	1.98E-11	21	92
GO:0030496	Midbody	CELLULAR	5.08E-10	2.67E-11	54	602
GO:0014704	Intercalated disc	CELLULAR	7.67E-10	4.11E-11	44	430
GO:0021510	Spinal cord development	BIOLOGICA L	7.68E-10	4.11E-11	53	591
GO:0008270	Zinc ion binding	MOLECULAR	1.27E-09	6.93E-11	90	1364
GO:0031646	Positive regulation of nervous system process	BIOLOGICAL	1.39E-09	7.61E-11	37	322
GO:0007628	Adult walking behavior	BIOLOGICAL	2.83E-09	1.60E-10	33	268
GO:0005096	Gtpase activator activity	MOLECULAR	3.13E-09	1.77E-10	44	452
GO:0070374	Positive regulation of ERK1 and ERK2 cascade	BIOLOGICAL	9.76E-09	5.79E-10	53	640
GO:0002066	Columnar/cuboidal epithelial cell development	BIOLOGICAL	1.81E-08	1.09E-09	56	712
GO:0021766	Hippocampus development	BIOLOGICAL	3.76E-08	2.35E-09	42	458
GO:0051260	Protein homooligomerization	BIOLOGICAL	5.49E-07	3.83E-08	42	509
GO:0005925	Focal adhesion	CELLULAR	1.33E-06	9.79E-08	47	629
GO:0030018	Z disc	CELLULAR	1.45E-06	1.07E-07	47	631
GO:0017048	Small gtpase binding	MOLECULAR	1.55E-06	1.15E-07	36	415
GO:0031965	Nuclear membrane	CELLULAR	2.01E-06	1.51E-07	51	723
GO:0005884	Actin filament	CELLULAR	6.89E-06	5.47E-07	40	525
GO:0051015	Actin filament binding	MOLECULAR	2.93E-05	2.54E-06	47	712
GO:0071560	Cellular response to transforming growth factor beta stimulus	BIOLOGICAL	4.22E-05	3.77E-06	51	813
GO:0007422	Peripheral nervous system development	BIOLOGICAL	5.34E-05	4.83E-06	36	493
GO:0043312	Neutrophil degranulation	BIOLOGICAL	7.74E-05	7.16E-06	48	765

GO:0000790	Chromatin	CELLULAR	9.11E-05	8.53E-06	80	1541
GO:0061041	Regulation of wound healing	BIOLOGICAL	1.06E-04	1.01E-05	42	641
GO:0000981	DNA-binding transcription factor activity, RNA polymerase II-specific	MOLECULAR	1.21E-04	1.17E-05	82	1606
GO:0009897	External side of plasma membrane	CELLULAR	2.86E-04	2.94E-05	42	673
GO:0031625	Ubiquitin protein ligase binding	MOLECULAR	2.88E-04	2.97E-05	49	835
GO:0016607	Nuclear speck	CELLULAR	8.24E-04	9.26E-05	50	901
GO:0001889	Liver development	BIOLOGICAL	0.001044448	1.21E-04	45	791
GO:0022408	Negative regulation of cell-cell adhesion	BIOLOGICAL	0.001213708	1.42E-04	37	609
GO:0072089	Stem cell proliferation	BIOLOGICAL	0.001451652	1.72E-04	41	709
GO:0045995	Regulation of embryonic development	BIOLOGICAL	0.001866748	2.27E-04	43	767
GO:0032355	Response to estradiol	BIOLOGICAL	0.00241735	3.01E-04	43	778
GO:0005525	GTP binding	MOLECULAR	0.003365324	4.33E-04	36	624
GO:0043161	Proteasome-mediated ubiquitin-dependent protein catabolic process	BIOLOGICAL	0.005016948	6.76E-04	46	886
GO:0001822	Kidney development	BIOLOGICAL	0.005537116	7.54E-04	63	1329
GO:0006954	Inflammatory response	BIOLOGICAL	0.005894901	8.08E-04	78	1734
GO:0042742	Defense response to bacterium	BIOLOGICAL	0.0065032	9.07E-04	39	725
GO:0045861	Negative regulation of proteolysis	BIOLOGICAL	0.006844754	9.59E-04	40	752
GO:0045444	Fat cell differentiation	BIOLOGICAL	0.008416452	0.001209092	42	812
GO:0060348	Bone development	BIOLOGICAL	0.010864295	0.001624603	45	902
GO:0007565	Female pregnancy	BIOLOGICAL	0.018154425	0.002926209	40	803
GO:0051216	Cartilage development	BIOLOGICAL	0.049905938	0.009505135	41	893

Table S6. Top 100 enriched GO terms for the Midbrain region (MB) of *L. dimidiatus* during the interaction treatment.

GO ID	Go name	GO Category	FDR	P-Value	Nr Test	Nr Reference
GO:0003735	Structural constituent of ribosome	MOLECULAR	5.93E-14	1.97E-16	22	187
GO:0098978	Glutamatergic synapse	CELLULAR	1.07E-12	3.97E-15	65	2177
GO:0005524	ATP binding	MOLECULAR	4.71E-10	2.81E-12	62	2338
GO:0000184	Nuclear-transcribed mrna catabolic process, nonsense-mediated decay	BIOLOGICAL	5.45E-10	3.29E-12	15	115
GO:0019228	Neuronal action potential	BIOLOGICAL	6.64E-09	4.93E-11	18	227
GO:0005730	Nucleolus	CELLULAR	1.49E-08	1.17E-10	58	2307
GO:0010881	Regulation of cardiac muscle contraction by regulation of the release of sequestered calcium ion	BIOLOGICAL	1.74E-07	1.84E-09	13	130
GO:0006614	SRP-dependent cotranslational protein targeting to membrane	BIOLOGICAL	4.56E-07	5.14E-09	9	49
GO:0022625	Cytosolic large ribosomal subunit	CELLULAR	6.85E-07	8.06E-09	10	72
GO:0042788	Polysomal ribosome	CELLULAR	1.07E-06	1.32E-08	11	100
GO:0098685	Schaffer collateral - CA1 synapse	CELLULAR	1.38E-06	1.75E-08	25	649
GO:0099059	Integral component of presynaptic active zone membrane	CELLULAR	1.74E-06	2.28E-08	14	195
GO:0086046	Membrane depolarization during SA node cell action potential	BIOLOGICAL	3.30E-06	4.65E-08	9	65
GO:0001518	Voltage-gated sodium channel complex	CELLULAR	3.30E-06	4.65E-08	8	45
GO:0086091	Regulation of heart rate by cardiac conduction	BIOLOGICAL	3.75E-06	5.43E-08	14	210
GO:0043522	Leucine zipper domain binding	MOLECULAR	4.52E-06	6.62E-08	9	68
GO:0005516	Calmodulin binding	MOLECULAR	5.31E-06	7.92E-08	26	756
GO:0019083	Viral transcription	BIOLOGICAL	6.81E-06	1.05E-07	16	298
GO:0008022	Protein C-terminus binding	MOLECULAR	7.60E-06	1.20E-07	29	936
GO:0035725	Sodium ion transmembrane transport	BIOLOGICAL	7.60E-06	1.19E-07	20	474
GO:0031594	Neuromuscular junction	CELLULAR	9.50E-06	1.52E-07	23	627
GO:0005832	Chaperonin-containing T-complex	CELLULAR	1.00E-05	1.61E-07	5	9
GO:0048266	Behavioral response to pain	BIOLOGICAL	1.10E-05	1.79E-07	12	163

GO:0031402	Sodium ion binding	MOLECULAR	1.18E-05	1.93E-07	9	78
GO:0002199	Zona pellucida receptor complex	CELLULAR	1.44E-05	2.40E-07	5	10
GO:0086045	Membrane depolarization during AV node cell action potential	BIOLOGICAL	1.78E-05	3.04E-07	8	59
GO:0098688	Parallel fiber to Purkinje cell synapse	CELLULAR	1.88E-05	3.22E-07	14	245
GO:1904851	Positive regulation of establishment of protein localization to telomere	BIOLOGICAL	2.01E-05	3.46E-07	5	11
GO:0007628	Adult walking behavior	BIOLOGICAL	2.01E-05	3.48E-07	15	286
GO:0032212	Positive regulation of telomere maintenance via telomerase	BIOLOGICAL	3.66E-05	6.61E-07	8	66
GO:0060024	Rhythmic synaptic transmission	BIOLOGICAL	4.73E-05	9.00E-07	8	69
GO:0004674	Protein serine/threonine kinase activity	MOLECULAR	5.48E-05	1.05E-06	25	814
GO:0044325	Ion channel binding	MOLECULAR	6.19E-05	1.24E-06	21	605
GO:0033270	Paranode region of axon	CELLULAR	6.19E-05	1.23E-06	11	162
GO:0070126	Mitochondrial translational termination	BIOLOGICAL	8.51E-05	1.76E-06	8	76
GO:0099508	Voltage-gated ion channel activity involved in regulation of presynaptic membrane potential	MOLECULAR	8.51E-05	1.76E-06	8	76
GO:0015871	Choline transport	BIOLOGICAL	8.54E-05	1.77E-06	7	52
GO:0043194	Axon initial segment	CELLULAR	8.71E-05	1.81E-06	11	169
GO:0070125	Mitochondrial translational elongation	BIOLOGICAL	9.14E-05	1.92E-06	8	77
GO:1902514	Regulation of calcium ion transmembrane transport via high voltage-gated calcium channel	BIOLOGICAL	9.14E-05	1.92E-06	8	77
GO:1990454	L-type voltage-gated calcium channel complex	CELLULAR	1.15E-04	2.48E-06	7	55
GO:0099524	Postsynaptic cytosol	CELLULAR	1.19E-04	2.58E-06	9	109
GO:0009790	Embryo development	BIOLOGICAL	1.37E-04	3.02E-06	86	5325
GO:1901385	Regulation of voltage-gated calcium channel activity	BIOLOGICAL	1.39E-04	3.06E-06	11	179
GO:0003729	Mrna binding	MOLECULAR	1.45E-04	3.22E-06	22	699
GO:0086056	Voltage-gated calcium channel activity involved in AV node cell action potential	MOLECULAR	1.46E-04	3.25E-06	6	36
GO:0043198	Dendritic shaft	CELLULAR	1.57E-04	3.51E-06	16	394
GO:0019901	Protein kinase binding	MOLECULAR	1.82E-04	4.17E-06	50	2553
GO:0099703	Induction of synaptic vesicle exocytosis by positive regulation of presynaptic cytosolic calcium ion concentration	BIOLOGICAL	1.86E-04	4.30E-06	6	38

GO:0046684	Response to pyrethroid	BIOLOGICAL	1.86E-04	4.30E-06	6	38
GO:0098982	GABA-ergic synapse	CELLULAR	1.94E-04	4.52E-06	18	501
GO:0008332	Low voltage-gated calcium channel activity	MOLECULAR	2.06E-04	4.80E-06	5	21
GO:0030165	PDZ domain binding	MOLECULAR	2.30E-04	5.43E-06	19	560
GO:0045433	Male courtship behavior, veined wing generated song production	BIOLOGICAL	2.45E-04	5.85E-06	5	22
GO:0006364	Rrna processing	BIOLOGICAL	2.48E-04	5.95E-06	14	318
GO:0035264	Multicellular organism growth	BIOLOGICAL	2.74E-04	6.62E-06	24	848
GO:1900170	Negative regulation of glucocorticoid mediated signaling pathway	BIOLOGICAL	3.63E-04	9.10E-06	3	2
GO:0086059	Voltage-gated calcium channel activity involved SA node cell action potential	MOLECULAR	4.64E-04	1.19E-05	5	26
GO:0071236	Cellular response to antibiotic	BIOLOGICAL	4.80E-04	1.24E-05	8	101
GO:0098912	Membrane depolarization during atrial cardiac muscle cell action potential	BIOLOGICAL	5.01E-04	1.30E-05	6	47
GO:0061003	Positive regulation of dendritic spine morphogenesis	BIOLOGICAL	5.32E-04	1.39E-05	9	136
GO:0001540	Amyloid-beta binding	MOLECULAR	5.35E-04	1.40E-05	13	298
GO:0043204	Perikaryon	CELLULAR	5.42E-04	1.42E-05	23	830
GO:0099635	Voltage-gated calcium channel activity involved in positive regulation of presynaptic cytosolic calcium levels	MOLECULAR	5.50E-04	1.45E-05	6	48
GO:0098831	Presynaptic active zone cytoplasmic component	CELLULAR	5.67E-04	1.50E-05	7	74
GO:0060314	Regulation of ryanodine-sensitive calcium-release channel activity	BIOLOGICAL	6.42E-04	1.72E-05	8	106
GO:0047485	Protein N-terminus binding	MOLECULAR	7.57E-04	2.05E-05	17	509
GO:0008179	Adenylate cyclase binding	MOLECULAR	8.08E-04	2.22E-05	7	79
GO:0051402	Neuron apoptotic process	BIOLOGICAL	8.08E-04	2.22E-05	28	1167
GO:0099505	Regulation of presynaptic membrane potential	BIOLOGICAL	8.08E-04	2.22E-05	7	79
GO:0033268	Node of Ranvier	CELLULAR	8.15E-04	2.24E-05	9	145
GO:0098556	Cytoplasmic side of rough endoplasmic reticulum membrane	CELLULAR	9.06E-04	2.54E-05	5	31
GO:0005246	Calcium channel regulator activity	MOLECULAR	9.43E-04	2.66E-05	11	228
GO:0005762	Mitochondrial large ribosomal subunit	CELLULAR	9.48E-04	2.67E-05	6	54
GO:1904874	Positive regulation of telomerase RNA localization to Cajal body	BIOLOGICAL	0.001096851	3.14E-05	3	4

GO:0031145	Anaphase-promoting complex-dependent catabolic process	BIOLOGICAL	0.001103383	3.16E-05	8	116
GO:0097110	Scaffold protein binding	MOLECULAR	0.001151711	3.31E-05	16	476
GO:0050998	Nitric-oxide synthase binding	MOLECULAR	0.001161075	3.35E-05	8	117
GO:1905274	Regulation of modification of postsynaptic actin cytoskeleton	BIOLOGICAL	0.001189255	3.44E-05	7	85
GO:0010960	Magnesium ion homeostasis	BIOLOGICAL	0.001263561	3.69E-05	7	86
GO:0007613	Memory	BIOLOGICAL	0.00131908	3.87E-05	24	949
GO:1905030	Voltage-gated ion channel activity involved in regulation of postsynaptic membrane potential	MOLECULAR	0.00131908	3.88E-05	6	58
GO:0099523	Presynaptic cytosol	CELLULAR	0.001416688	4.20E-05	8	121
GO:0008627	Intrinsic apoptotic signaling pathway in response to osmotic stress	BIOLOGICAL	0.00142634	4.24E-05	6	59
GO:2001258	Negative regulation of cation channel activity	BIOLOGICAL	0.001431757	4.26E-05	10	198
GO:0003725	Double-stranded RNA binding	MOLECULAR	0.001542128	4.62E-05	11	243
GO:0048791	Calcium ion-regulated exocytosis of neurotransmitter	BIOLOGICAL	0.001612382	4.84E-05	7	90
GO:0001966	Thigmotaxis	BIOLOGICAL	0.001613025	4.85E-05	5	36
GO:0090666	Scarna localization to Cajal body	BIOLOGICAL	0.001648659	4.99E-05	3	5
GO:0022627	Cytosolic small ribosomal subunit	CELLULAR	0.001660672	5.03E-05	6	61
GO:0016322	Neuron remodeling	BIOLOGICAL	0.001959011	6.08E-05	10	207
GO:0019227	Neuronal action potential propagation	BIOLOGICAL	0.002010374	6.27E-05	7	94
GO:0008331	High voltage-gated calcium channel activity	MOLECULAR	0.002181174	6.87E-05	5	39
GO:0098696	Regulation of neurotransmitter receptor localization to postsynaptic specialization membrane	BIOLOGICAL	0.002240886	7.12E-05	8	131
GO:0005684	U2-type spliceosomal complex	CELLULAR	0.002240886	7.12E-05	8	131
GO:0097470	Ribbon synapse	CELLULAR	0.002280132	7.27E-05	9	170
GO:0001740	Barr body	CELLULAR	0.00232147	7.42E-05	3	6

Table S7. Top 100 enriched GO terms for the Hindbrain region (HB) of *L. dimidiatus* during the interaction treatment.

GO ID	Go name	GO Category	FDR	P-Value	Nr Test	Nr Reference
GO:0098978	Glutamatergic synapse	CELLULAR	2.97E-65	1.45E-67	377	1865
GO:0098982	GABA-ergic synapse	CELLULAR	1.16E-29	1.78E-31	115	404
GO:0099061	Integral component of postsynaptic density membrane	CELLULAR	8.70E-29	1.38E-30	98	303
GO:0043204	Perikaryon	CELLULAR	1.28E-26	2.18E-28	149	704
GO:1904315	Transmitter-gated ion channel activity involved in regulation of postsynaptic membrane potential	MOLECULAR	4.83E-23	9.32E-25	52	96
GO:0031594	Neuromuscular junction	CELLULAR	5.89E-21	1.22E-22	115	535
GO:0043524	Negative regulation of neuron apoptotic process	BIOLOGICAL	2.17E-18	5.04E-20	114	576
GO:0000381	Regulation of alternative mrna splicing, via spliceosome	BIOLOGICAL	4.29E-17	1.10E-18	51	137
GO:0098685	Schaffer collateral - CA1 synapse	CELLULAR	6.45E-17	1.68E-18	109	565
GO:0001540	Amyloid-beta binding	MOLECULAR	4.50E-16	1.26E-17	66	245
GO:0099583	Neurotransmitter receptor activity involved in regulation of postsynaptic cytosolic calcium ion concentration	MOLECULAR	5.70E-16	1.60E-17	24	20
GO:0032281	AMPA glutamate receptor complex	CELLULAR	8.37E-16	2.38E-17	36	68
GO:0051602	Response to electrical stimulus	BIOLOGICAL	1.23E-15	3.53E-17	65	244
GO:0051117	Atpase binding	MOLECULAR	1.48E-15	4.30E-17	75	318
GO:0009897	External side of plasma membrane	CELLULAR	1.65E-15	4.80E-17	110	605
GO:2000310	Regulation of NMDA receptor activity	BIOLOGICAL	2.81E-15	8.36E-17	55	181
GO:0043083	Synaptic cleft	CELLULAR	2.90E-15	8.65E-17	44	114
GO:0051965	Positive regulation of synapse assembly	BIOLOGICAL	5.38E-15	1.63E-16	80	366
GO:0016607	Nuclear speck	CELLULAR	6.38E-15	1.93E-16	132	819
GO:0032839	Dendrite cytoplasm	CELLULAR	6.70E-15	2.03E-16	47	135
GO:0008022	Protein C-terminus binding	MOLECULAR	8.59E-15	2.62E-16	133	832
GO:0043198	Dendritic shaft	CELLULAR	1.55E-14	4.77E-16	75	335
GO:0098688	Parallel fiber to Purkinje cell synapse	CELLULAR	4.76E-14	1.50E-15	56	203

GO:0004971	AMPA glutamate receptor activity	MOLECULAR	9.53E-14	3.07E-15	18	10
GO:0031489	Myosin V binding	MOLECULAR	1.51E-13	4.93E-15	29	50
GO:0044308	Axonal spine	CELLULAR	2.91E-13	9.63E-15	19	14
GO:0006406	Mrna export from nucleus	BIOLOGICAL	3.56E-13	1.19E-14	45	141
GO:0030165	PDZ domain binding	MOLECULAR	5.41E-13	1.83E-14	90	489
GO:0060992	Response to fungicide	BIOLOGICAL	8.24E-13	2.81E-14	28	50
GO:0098686	Hippocampal mossy fiber to CA3 synapse	CELLULAR	9.85E-13	3.38E-14	58	236
GO:0015277	Kainate selective glutamate receptor activity	MOLECULAR	1.11E-12	3.82E-14	20	19
GO:0032983	Kainate selective glutamate receptor complex	CELLULAR	1.11E-12	3.82E-14	20	19
GO:0071418	Cellular response to amine stimulus	BIOLOGICAL	1.22E-12	4.20E-14	19	16
GO:0071359	Cellular response to dsrna	BIOLOGICAL	1.88E-12	6.57E-14	30	62
GO:0035641	Locomotory exploration behavior	BIOLOGICAL	0.005441715	6.67E-04	16	83
GO:0005912	Adherens junction	CELLULAR	3.59E-12	1.27E-13	92	526
GO:0001662	Behavioral fear response	BIOLOGICAL	6.28E-12	2.28E-13	54	219
GO:0032809	Neuronal cell body membrane	CELLULAR	7.20E-12	2.62E-13	49	184
GO:0071230	Cellular response to amino acid stimulus	BIOLOGICAL	2.64E-11	9.87E-13	59	267
GO:0007218	Neuropeptide signaling pathway	BIOLOGICAL	3.04E-11	1.14E-12	43	151
GO:0005789	Endoplasmic reticulum membrane	CELLULAR	5.10E-11	1.94E-12	200	1660
GO:0032591	Dendritic spine membrane	CELLULAR	6.50E-11	2.49E-12	23	39
GO:0098843	Postsynaptic endocytic zone	CELLULAR	7.68E-11	2.97E-12	19	23
GO:0047485	Protein N-terminus binding	MOLECULAR	1.10E-10	4.28E-12	79	447
GO:0007616	Long-term memory	BIOLOGICAL	1.36E-10	5.36E-12	59	280
GO:0021766	Hippocampus development	BIOLOGICAL	1.51E-10	6.00E-12	76	424
GO:0031681	G-protein beta-subunit binding	MOLECULAR	1.59E-10	6.31E-12	25	51
GO:1905606	Regulation of presynapse assembly	BIOLOGICAL	1.88E-10	7.52E-12	46	183
GO:0055038	Recycling endosome membrane	CELLULAR	2.30E-10	9.25E-12	48	199
GO:1905852	Positive regulation of backward locomotion	BIOLOGICAL	3.21E-10	1.32E-11	15	12

GO:0097110	Scaffold protein binding	MOLECULAR	4.54E-10	1.89E-11	74	418
GO:0008179	Adenylate cyclase binding	MOLECULAR	5.03E-10	2.11E-11	26	60
GO:0098981	Cholinergic synapse	CELLULAR	5.55E-10	2.34E-11	24	50
GO:2001224	Positive regulation of neuron migration	BIOLOGICAL	1.41E-09	6.12E-11	31	93
GO:0048169	Regulation of long-term neuronal synaptic plasticity	BIOLOGICAL	1.44E-09	6.25E-11	44	182
GO:0001965	G-protein alpha-subunit binding	MOLECULAR	1.92E-09	8.46E-11	41	162
GO:1905850	Positive regulation of forward locomotion	BIOLOGICAL	2.18E-09	9.63E-11	11	4
GO:0048148	Behavioral response to cocaine	BIOLOGICAL	8.76E-05	6.88E-06	21	88
GO:0046982	Protein heterodimerization activity	MOLECULAR	2.23E-09	9.85E-11	108	760
GO:0051018	Protein kinase A binding	MOLECULAR	3.92E-09	1.76E-10	37	138
GO:0051968	Positive regulation of synaptic transmission, glutamatergic	BIOLOGICAL	1.23E-08	5.80E-10	34	124
GO:1990830	Cellular response to leukemia inhibitory factor	BIOLOGICAL	1.27E-08	6.04E-10	68	401
GO:1902476	Chloride transmembrane transport	BIOLOGICAL	1.99E-08	9.57E-10	39	163
GO:0050733	RS domain binding	MOLECULAR	2.06E-08	9.89E-10	12	9
GO:0099151	Regulation of postsynaptic density assembly	BIOLOGICAL	2.49E-08	1.21E-09	28	88
GO:0005925	Focal adhesion	CELLULAR	2.59E-08	1.26E-09	87	589
GO:0099507	Ligand-gated ion channel activity involved in regulation of presynaptic membrane potential	MOLECULAR	2.64E-08	1.28E-09	17	28
GO:0001975	Response to amphetamine	BIOLOGICAL	2.65E-08	1.29E-09	39	165
GO:0005246	Calcium channel regulator activity	MOLECULAR	2.77E-08	1.35E-09	43	196
GO:0042803	Protein homodimerization activity	MOLECULAR	3.77E-08	1.85E-09	204	1861
GO:0070374	Positive regulation of ERK1 and ERK2 cascade	BIOLOGICAL	3.98E-08	1.96E-09	88	605
GO:0035176	Social behavior	BIOLOGICAL	0.005381877	6.58E-04	30	214
GO:0048266	Behavioral response to pain	BIOLOGICAL	4.63E-05	3.42E-06	29	146
GO:1900454	Positive regulation of long-term synaptic depression	BIOLOGICAL	4.27E-08	2.11E-09	24	66
GO:0042711	Maternal behavior	BIOLOGICAL	0.00313781	3.57E-04	19	103
GO:0099566	Regulation of postsynaptic cytosolic calcium ion concentration	BIOLOGICAL	4.27E-08	2.11E-09	24	66
GO:0008076	Voltage-gated potassium channel complex	CELLULAR	4.57E-08	2.26E-09	46	224

GO:0061744	Motor behavior	BIOLOGICAL	0.005986758	7.43E-04	11	44
GO:0012507	ER to Golgi transport vesicle membrane	CELLULAR	5.46E-08	2.73E-09	21	50
GO:0007628	Adult walking behavior	BIOLOGICAL	7.37E-06	4.79E-07	44	257
GO:0007605	Sensory perception of sound	BIOLOGICAL	9.12E-06	6.01E-07	97	794
GO:0019865	Immunoglobulin binding	MOLECULAR	6.20E-08	3.11E-09	18	35
GO:0048025	Negative regulation of mrna splicing, via spliceosome	BIOLOGICAL	6.77E-08	3.42E-09	24	68
GO:0031698	Beta-2 adrenergic receptor binding	MOLECULAR	7.95E-08	4.04E-09	22	57
GO:0007625	Grooming behavior	BIOLOGICAL	9.02E-04	8.88E-05	18	83
GO:0030027	Lamellipodium	CELLULAR	8.52E-08	4.34E-09	102	758
GO:1904837	Beta-catenin-TCF complex assembly	BIOLOGICAL	9.28E-08	4.77E-09	21	52
GO:0045838	Positive regulation of membrane potential	BIOLOGICAL	9.60E-08	4.94E-09	26	82
GO:0010226	Response to lithium ion	BIOLOGICAL	1.14E-07	5.91E-09	34	138
GO:1902287	Semaphorin-plexin signaling pathway involved in axon guidance	BIOLOGICAL	1.19E-07	6.20E-09	14	19
GO:0048149	Behavioral response to ethanol	BIOLOGICAL	0.005381877	6.58E-04	20	118
GO:0034332	Adherens junction organization	BIOLOGICAL	1.76E-07	9.32E-09	50	270
GO:0045163	Clustering of voltage-gated potassium channels	BIOLOGICAL	2.56E-07	1.37E-08	12	13
GO:0044307	Dendritic branch	CELLULAR	2.90E-07	1.55E-08	14	21
GO:0007614	Short-term memory	BIOLOGICAL	2.94E-07	1.57E-08	33	137
GO:0008270	Zinc ion binding	MOLECULAR	3.55E-07	1.92E-08	150	1304
GO:0007157	Heterophilic cell-cell adhesion via plasma membrane cell adhesion molecules	BIOLOGICAL	3.60E-07	1.94E-08	32	131

Table S8. Enriched GO terms for the Forebrain region (FB) of *A. leucosternon* during the interaction treatment.

GO ID	GO Name	GO Category	FDR	P-Value	Nr Test	Nr Reference
GO:0015669	Gas transport	BIOLOGICAL	0.001508	3.85E-07	4	38
GO:0015671	Oxygen transport	BIOLOGICAL	0.013584	4.63E-06	3	20
GO:0048018	Receptor ligand activity	MOLECULAR	2.44E-06	2.08E-10	11	518
GO:0030546	Signalling receptor activator activity	MOLECULAR	3.07E-06	3.92E-10	11	551
GO:0030545	Receptor regulator activity	MOLECULAR	3.76E-06	6.41E-10	11	578
GO:0005179	Hormone activity	MOLECULAR	9.53E-11	4.06E-15	10	122
GO:0031720	Haptoglobin binding	MOLECULAR	0.001046	2.23E-07	3	6
GO:0005344	Oxygen carrier activity	MOLECULAR	0.013584	4.63E-06	3	20

Table S9. Enriched GO terms for the Midbrain region (MB) of *A. leucosternon* during the interaction treatment.

GO ID	GO Name	GO Category	FDR	P-Value	Nr Test	Nr Reference
GO:0032482	Rab protein signal transduction	BIOLOGICAL	0.027408	1.17E-06	4	74

Table S10. Enriched GO terms for the Hindbrain region (HB) of *A. leucosternon* during the interaction treatment.

GO ID	GO Name	GO Category	FDR	P-Value	Nr Test	Nr Reference
GO:0009987	Cellular process	BIOLOGICAL	0.043421	1.66E-05	106	24300
GO:0065007	Biological regulation	BIOLOGICAL	0.015566	3.32E-06	97	20817
GO:0050789	Regulation of biological process	BIOLOGICAL	0.009169	1.56E-06	96	20222
GO:0006259	DNA metabolic process	BIOLOGICAL	0.0259	8.83E-06	22	2312
GO:0006281	DNA repair	BIOLOGICAL	0.019758	5.89E-06	14	980
GO:0072698	Protein localization to microtubule cytoskeleton	BIOLOGICAL	0.00591	2.52E-07	8	182
GO:0044380	Protein localization to cytoskeleton	BIOLOGICAL	0.009151	7.91E-07	8	213
GO:0005488	Binding	MOLECULAR	0.017505	4.47E-06	107	24069
GO:0005515	Protein binding	MOLECULAR	0.009151	1.17E-06	104	22583

Table S11. Immediate early genes (IEG) differentially expression in the three brain regions of *L. dimidiatus* during the interaction treatment. Column “SeqName” represents the transcript sequence obtained with their respective Trinity ID. Grouped columns by “Forebrain”, “Midbrain” and “Hindbrain” contain the Differential expression analysis (DESeq2) results for each region where the values of log2fold changes are represented by positive (upregulated expression) or negative (downregulated expression) values, and its significance highlighted in **bold** (padj <0.05). Grouped columns by “Gene Annotation”, represent the gene annotation for each transcript (Swissprot-blast), their respective Gene symbol and its full name.

	**Forebrain**				**Midbrain**				**Hindbrain**				**Gene Annotation**	
**SeqName**	**Base Mean**	**log2Fold Change**	**padj**	**SIG**	**Base Mean**	**log2Fold Change**	**padj**	**SIG**	**Base Mean**	**log2Fold Change**	**padj**	**SIG**	**Gene symbol**	**Gene name**
TRINITY_DN1891_c0_g2	27.73	-0.34	0.47		167.02	0.03	0.94		28.31	-1.33	0.00	**sig**	CEBPA	CCAAT/enhancer-binding protein alpha
TRINITY_DN854_c6_g1	38.13	-1.09	0.01	**sig**	56.24	-0.89	0.02	**sig**	50.83	-0.09	0.89		CREB	CREB3 regulatory factor
TRINITY_DN10607_c0_g1	50.52	-1.88	0.02	**sig**	66.18	-1.40	0.00		20.11	-2.40	0.00	**sig**	DUS10	Dual specificity protein phosphatase 10
TRINITY_DN10801_c2_g1	44.93	1.28	0.04	**sig**	56.34	0.48	0.37		34.26	-0.02	0.99		DUS14	Dual specificity protein phosphatase 14
TRINITY_DN103_c3_g1	1816.44	0.38	0.23		1834.16	0.34	0.33		2723.76	0.80	0.02	**sig**	DUS16	Dual specificity protein phosphatase 16
TRINITY_DN103_c3_g1	90.87	-0.90	0.01	**sig**	47.19	-0.40	0.15		35.51	-0.58	0.20		DUS7	Dual specificity protein phosphatase 7
TRINITY_DN1891_c0_g1	227.03	0.49	0.03	**sig**	1074.49	0.07	0.87		207.51	-0.92	0.06		FOS	Proto-oncogene c-Fos
TRINITY_DN1737_c0_g1	491.28	0.55	0.08		2302.98	0.13	0.79		713.52	-0.71	0.04	**sig**	FOS	Proto-oncogene c-Fos
TRINITY_DN1353_c0_g1	597.31	-0.60			50.59	-0.06	0.91		28.79	-0.74	0.05	**sig**	GNRR2	Gonadotropin-releasing hormone II receptor
TRINITY_DN6851_c7_g1	1766.47	-0.22	0.47		1012.84	-0.04	0.92		333.28	-0.77	0.03	**sig**	HOME1	Homer protein homolog 1

TRINITY_DN14358_c0_g1	1105.92	-0.47	0.04	**sig**	1857.76	-0.34	0.06		1103.16	-0.37	0.05	**sig**	JIP2	C-Jun-amino-terminal kinase-interacting protein 2
TRINITY_DN2796_c1_g2	623.31	0.09	0.57		1101.98	0.21	0.56		905.43	0.60	0.00	**sig**	JIP3	C-Jun-amino-terminal kinase-interacting protein 3
TRINITY_DN3883_c0_g2	130.40	0.47	0.11		437.49	0.41	0.29		553.46	0.65	0.03	**sig**	JUN	Jun proto-oncogene, AP-1 transcription factor subunit
TRINITY_DN8120_c0_g2	440.59	0.58	0.02	**sig**	166.72	0.88	0.05		251.50	1.01	0.01	**sig**	KLF11	Krueppel-like factor 11
TRINITY_DN41677_c0_g1	268.99	-0.61	0.06		154.48	0.02	0.95		41.61	-0.84	0.03	**sig**	KLF12	Krueppel-like factor 12
TRINITY_DN40623_c0_g1	48.24	-0.70	0.03	**sig**	60.22	-0.46	0.06		50.02	0.06	0.93		klf12b	Krueppel-like factor 12
TRINITY_DN7120_c0_g2	86.14	-0.30	0.32		112.40	-0.26	0.09		80.12	-0.63	0.02	**sig**	KLF15	Krueppel-like factor 15
TRINITY_DN2160_c1_g1	230.66	-0.62	0.04	**sig**	657.02	-0.48	0.04	**sig**	892.23	0.22	0.65		KLF5	Krueppel-like factor 5
TRINITY_DN1251_c0_g1	1227.73	0.50	0.01	**sig**	1943.42	0.31	0.24		503.79	0.53	0.03	**sig**	MCL1	Induced myeloid leukemia cell differentiation protein Mcl-1 homolog
TRINITY_DN2637_c0_g3	58.09	0.66	0.11		228.20	0.57	0.00	**sig**	44.23	-1.15	0.01	**sig**	MCL1B	Protein L-Myc-1b
TRINITY_DN18783_c0_g1	47.19	0.00	1.00		106.17	-0.51	0.22		39.63	-1.31	0.02	**sig**	NPAS3	Neuronal PAS domain-containing protein 3
TRINITY_DN16301_c0_g1	514.07	-0.57	0.02	**sig**	811.84	-0.21	0.59		1230.19	0.09	0.90		NPS4L	Neuronal PAS domain-containing protein 4-like
TRINITY_DN12415_c0_g1	47.03	-0.21	0.64		57.74	-0.66	0.12		61.81	-0.72	0.02	**sig**	RGS19	Regulator of G-protein signaling 19
TRINITY_DN1383_c1_g1	169.89	-0.11	0.66		306.47	0.12	0.57		98.57	-1.15	0.00	**sig**	RGS6	Regulator of G-protein signaling 6
TRINITY_DN19893_c0_g2	410.99	0.23	0.33		342.26	0.08	0.91		327.70	0.60	0.00	**sig**	RGS9	Regulator of G-protein signaling 9
TRINITY_DN12104_c0_g2	964.46	0.33	0.04	**sig**	1373.17	0.02	0.93		1284.28	0.23	0.51		RHEB	GTP-binding protein Rheb
TRINITY_DN3980_c0_g1	345.59	0.43	0.43		242.74	0.24	0.62		135.14	-1.82	0.03	**sig**	RHES	GTP-binding protein Rhes

TRINITY_DN1654_c0_g3	173.74	-0.59	0.05	**sig**	579.64	-0.45	0.19		281.47	-0.35	0.24		SBK1	Serine/threonine-protein kinase SBK1
TRINITY_DN666_c0_g1	125.49	-0.40	0.07		208.49	-0.37	0.28		104.46	-1.03	0.00	**sig**	SBK1	Serine/threonine-protein kinase SBK1
TRINITY_DN8036_c0_g1	46.47	-0.81	0.02	**sig**	71.75	-0.62	0.08		58.70	-0.16	0.81		SOCS5	Suppressor of cytokine signaling 5
TRINITY_DN8036_c0_g2	92.54	-0.58	0.03	**sig**	149.42	-0.22	0.58		94.55	-0.40	0.33		TOB2	Protein Tob2

Table S12. Dopamine and behavioural hormones (Isotocin, Estrogen and Progesterone) genes differentially expressed in the three brain regions of *L. dimidiatus* during the interaction treatment. Column "SeqName" represents the transcript sequence obtained with their respective Trinity ID. Grouped columns by "Forebrain", "Midbrain" and "Hindbrain" contain the Differential expression analysis (DESeq2) results for each region where the values of log2fold changes are represented by positive (upregulated expression) or negative (downregulated expression) values, and its significance highlighted in **bold** (padj <0.05). Grouped columns by "Gene Annotation", represent the gene annotation for each transcript (Swissprot-blast), their respective Gene symbol and its full name

	**Forebrain**				**Midbrain**				**Hindbrain**				**Gene Annotation**	
**SeqName**	**Base Mean**	**log2Fold Change**	**padj**	**SIG**	**Base Mean**	**log2Fold Change**	**padj**	**SIG**	**Base Mean**	**log2Fold Change**	**padj**	**SIG**	**Gene symbol**	**Gene name**
TRINITY_DN3892_c0_g1	135.03	-0.96	0.01	**sig**	215.15	-0.55	0.04	**sig**	340.43	0.12	0.82		SV2C	Synaptic vesicle glycoprotein 2C
TRINITY_DN9585_c2_g2	4.70	-0.76	--			1.03	--		33.39	-1.86	0.02	**sig**	DDC	Aromatic-L-amino-acid decarboxylase
TRINITY_DN10322_c0_g1	46.88	-0.70	0.16		69.59	-0.65	0.13		76.12	-1.95	0.00	**sig**	SV2C	Synaptic vesicle glycoprotein 2C
TRINITY_DN526_c0_g1	44.34	-0.53	0.20		66.15	-0.47	0.36		17.05	-1.58	0.05	**sig**	DRD1L	(1)-like dopamine receptor
TRINITY_DN17756_c0_g1	60.96	-0.34	0.53		146.79	0.04	0.90		38.62	-2.08	0.00	**sig**	DRD2L	(2)-like dopamine receptor
TRINITY_DN10696_c0_g1	16.08	0.30	--		64.67	-0.06	0.88		12.94	-2.46	0.01	**sig**	DRD5	(1B) dopamine receptor
TRINITY_DN32088_c0_g1	19.37	-0.22	--		3.76	0.66	--		55.74	-1.63	0.00	**sig**	TY3H	Tyrosine 3-monooxygenase
TRINITY_DN47081_c0_g2	26.51	0.55	0.65		28.16	-0.08	--		143.08	-1.28	0.05	**sig**	DOPO	Dopamine beta-hydroxylase
TRINITY_DN21729_c0_g1		0.49	--			1.14	--		22.68	-1.43	0.04	**sig**	ERR3	Estrogen-related receptor gamma
TRINITY_DN16335_c0_g1		1.24	--		64.06	-0.02	0.97		29.13	-2.27	0.00	**sig**	ITR	Isotocin receptor
TRINITY_DN8359_c1_g2	339.48	-0.41	0.03	**sig**	420.66	-0.08	0.82		334.81	-0.17	0.63		PRGR	Membrane-associated progesterone receptor component 2

Table S13. Glutamatergic-synapse pathway genes differentially expressed in the three brain regions of *L. dimidiatus* during the interaction treatment. Column "SeqName" represents the transcript sequence obtained with their respective Trinity ID. Grouped columns by "Forebrain", "Midbrain" and "Hindbrain" contain the Differential expression analysis (DESeq2) results for each region where the values of log2fold changes are represented by positive (upregulated expression) or negative (downregulated expression) values, and its significance highlighted in **bold** (padj <0.05). Grouped columns by "Gene Annotation", represent the gene annotation for each transcript (Swissprot-blast), their respective Gene symbol and its full name

	Forebrain				Midbrain				Hindbrain				Gene Annotation	
**SeqName**	**Base Mean**	**log2 Fold Change**	**padj**	**SIG**	**Base Mean**	**log2 Fold Change**	**padj**	**SIG**	**Base Mean**	**log2 Fold Change**	**padj**	**SIG**	**Gene symbol**	**Gene name**
TRINITY_DN13387_c0_g2	20.81	-0.69	0.00		154.91	-0.60	0.01	**sig**	182.28	0.68	0.34		ADCY2	Adenylate cyclase type 2
TRINITY_DN967_c0_g2	56.24	-1.01	0.03	**sig**	55.93	-0.57	0.14		31.92	-1.37	0.00	**sig**	ADCY3	Adenylate cyclase type 3
TRINITY_DN8014_c0_g1	45.37	-0.86	0.09		--	-0.49	--		23.52	-2.44	0.00	**sig**	CA2D1	Voltage-dependent calcium channel subunit alpha-2/delta-1
TRINITY_DN211_c0_g1	357.00	-0.84	0.05	**sig**	263.59	-0.61	0.04	**sig**	223.14	0.31	0.54		CA2D2	Voltage-dependent calcium channel subunit alpha-2/delta-2
TRINITY_DN2_c1_g1	27.10	-0.11	0.86		284.94	-0.54	0.22		35.43	-2.17	0.02	**sig**	CABP7	Calcium-binding protein 7
TRINITY_DN31636_c0_g1	92.34	-0.95	0.02	**sig**	283.04	-0.69	0.00	**sig**	197.08	0.11	0.87		CAC1A	Voltage-dependent P/Q-type calcium channel subunit alpha-1A
TRINITY_DN11896_c0_g1	56.21	-0.73	0.15		63.82	-0.51	0.21		22.06	-1.59	0.01	**sig**	CAC1B	Probable voltage-dependent N-type calcium channel subunit alpha-1B
TRINITY_DN1384_c0_g3	73.52	-1.40	0.02	**sig**	120.42	-0.81	0.00	**sig**	163.97	0.49	0.45		CAC1C	Voltage-dependent L-type calcium channel subunit alpha-1C
TRINITY_DN1817_c0_g2	95.99	-0.81	0.01	**sig**	150.80	-0.57	0.02	**sig**	80.48	-0.17	0.73		CAC1D	Voltage-dependent L-type calcium channel subunit alpha-1D
TRINITY_DN12178_c0_g1	120.18	-1.48	0.00	**sig**	60.44	-0.97	0.02	**sig**	26.48	-1.22	0.03	**sig**	CAC1G	Voltage-dependent T-type calcium channel subunit alpha-1G
TRINITY_DN4061_c0_g2	187.97	-0.48	0.04	**sig**	404.88	-0.34	0.28		315.69	0.58	0.15		CAC1H	Voltage-dependent T-type calcium channel subunit alpha-1H
TRINITY_DN4177_c0_g1	53.55	-0.60	0.02	**sig**	123.76	-0.56	0.02	**sig**	94.08	0.27	0.45		CAC1I	Voltage-dependent T-type calcium channel subunit alpha-1I

TRINITY_DN10121_c0_g1	944.83	-0.47	0.04	**sig**	570.88	-0.05	0.78		466.21	0.37	0.19		CACB2	Voltage-dependent L-type calcium channel subunit beta-2
TRINITY_DN707_c0_g1	1181.41	0.06	0.65		2884.88	0.08	0.59		2424.04	0.44	0.00	**sig**	CASKA	calcium/calmodulin dependent serine protein kinase
TRINITY_DN2808_c3_g1	463.27	-0.01	0.97		582.12	0.17	0.46		450.04	0.65	0.02	**sig**	CCG2	Voltage-dependent calcium channel gamma-2 subunit
TRINITY_DN700_c0_g2	379.01	-1.20	0.04	**sig**	47.84	0.21	0.81		55.54	-1.07	0.04	**sig**	CCG4	Voltage-dependent calcium channel gamma-4 subunit
TRINITY_DN3729_c0_g1	205.49	-0.97	0.01	**sig**	--	0.31	--		22.41	-0.24	0.80		CCG5	Voltage-dependent calcium channel gamma-5 subunit
TRINITY_DN5342_c2_g1	337.36	-1.30	0.00	**sig**	49.24	-0.49	0.32		27.86	-2.80	0.00	**sig**	CCG8	Voltage-dependent calcium channel gamma-8 subunit
TRINITY_DN5466_c0_g1	298.43	-0.58	0.02	**sig**	724.14	-0.26	0.24		596.21	0.25	0.64		CPLX2	Calcium/calmodulin-dependent protein kinase type 1
TRINITY_DN2091_c1_g3	44.15	-1.03	0.01	**sig**	101.58	-0.57	0.25		85.01	0.04	0.97		CSKP	Calcium/calmodulin-dependent protein kinase type 1D
TRINITY_DN1059_c5_g1	126.92	-0.94	0.01	**sig**	178.73	-0.52	0.24		105.33	-1.64	0.00	**sig**	EAA3	Excitatory amino acid transporter 3
TRINITY_DN4519_c0_g1	428.18	-0.59	0.01	**sig**	444.93	-0.36	0.04	**sig**	361.66	0.08	0.86		GABR1	Gamma-aminobutyric acid type B receptor subunit 1
TRINITY_DN8889_c0_g1	334.21	-0.43	0.04	**sig**	354.12	-0.25	0.49		237.06	-0.08	0.81		GABR2	Gamma-aminobutyric acid type B receptor subunit 2
TRINITY_DN25773_c0_g1	6798.07	0.49	0.02	**sig**	7512.77	0.26	0.32		7154.72	0.29	0.25		GBLP	Guanine nucleotide-binding protein subunit beta-2-like 1
TRINITY_DN19226_c0_g1	100.32	-0.83	0.03	**sig**	101.31	-0.42	0.34		32.76	-2.76	0.00	**sig**	GBRB4	Gamma-aminobutyric acid receptor subunit beta-4
TRINITY_DN1643_c1_g2	26.67	-0.79	0.12		72.53	-0.39	0.49		21.84	-2.93	0.00	**sig**	GBRG1	Gamma-aminobutyric acid receptor subunit gamma-1
TRINITY_DN3317_c0_g2	331.92	-0.87	0.01	**sig**	512.06	-0.11	0.82		34.28	-1.76	0.03	**sig**	GBRP	Gamma-aminobutyric acid receptor subunit pi
TRINITY_DN7674_c0_g1	374.11	0.30	0.56		126.29	-0.34	0.57		26.18	-2.50	0.01	**sig**	GBRR1	Gamma-aminobutyric acid receptor subunit rho-1
TRINITY_DN23114_c0_g1	--	-0.89	--		61.47	-0.77	0.17		10.20	-3.95	0.00	**sig**	GBRR2	Gamma-aminobutyric acid receptor subunit rho-2
TRINITY_DN91_c0_g1	82.78	-0.65	0.02	**sig**	134.72	-0.44	0.04	**sig**	118.51	0.02	0.97		GCR	Glucocorticoid receptor
TRINITY_DN75221_c0_g5	77.07	0.02	0.95		92.71	-0.11	0.70		38.01	-1.71	0.00	**sig**	GCYA1	Guanylate cyclase soluble subunit alpha-1

TRINITY_DN3851_c0_g1	170.10	-0.55	0.04	**sig**	122.56	-0.23	0.65		44.54	-0.98	0.03	**sig**	GCYA2	Guanylate cyclase soluble subunit alpha-2
TRINITY_DN11740_c0_g1	643.97	0.11	0.60		487.95	0.08	0.79		360.85	0.52	0.01	**sig**	GCYB1	Guanylate cyclase soluble subunit beta-1
TRINITY_DN15288_c0_g1	--	-0.54	--		--	-0.62	--		42.80	-2.40	0.00	**sig**	GLRA1	Glycine receptor subunit alphaZ1
TRINITY_DN15288_c0_g4	48.12	0.23	0.67		105.12	0.08	0.89		30.84	-1.90	0.02	**sig**	GLRA2	Glycine receptor subunit alpha-2
TRINITY_DN840_c0_g2	67.33	0.49	0.35		117.62	0.36	0.32		33.03	-1.21	0.04	**sig**	GLRA4	Glycine receptor subunit alpha-4
TRINITY_DN12051_c1_g2	43.56	-0.24	0.69		93.57	-0.30	0.65		95.75	-2.31	0.00	**sig**	GLRB	Glycine receptor subunit beta
TRINITY_DN965_c0_g1	848.22	-0.05	0.80		801.62	0.18	0.58		840.29	0.76	0.01	**sig**	GLSK	Glutaminase kidney isoform, mitochondrial
TRINITY_DN390_c0_g1	78.53	-0.22	0.54		125.20	-0.12	0.66		143.73	0.68	0.00	**sig**	GNA13	Guanine nucleotide-binding protein subunit alpha-13
TRINITY_DN390_c0_g3	373.81	-0.20	0.51		521.21	-0.03	0.90		371.32	-0.34	0.04	**sig**	GNAI1	Guanine nucleotide-binding protein G(i) subunit alpha-1
TRINITY_DN138_c0_g1	549.24	0.76	0.00	**sig**	662.56	0.42	0.01	**sig**	726.42	0.15	0.67		GNAI2	Guanine nucleotide-binding protein G(i) subunit alpha-2
TRINITY_DN8643_c0_g1	90.78	-0.48	0.03	**sig**	129.63	0.03	0.95		119.10	0.53	0.20		GNAQ	ELL-associated factor 1
TRINITY_DN8168_c0_g1	1671.73	0.40	0.00	**sig**	2291.52	0.21	0.24		1554.03	0.19	0.37		GNB5A	Guanine nucleotide-binding protein subunit beta-5a
TRINITY_DN4500_c0_g1	262.49	-0.40	0.07		314.31	-0.40	0.02	**sig**	255.06	-0.13	0.58		GNL1	Guanine nucleotide-binding protein-like 1 OS
TRINITY_DN8490_c0_g1	128.86	-1.22	0.03	**sig**	128.75	-0.41	0.12		44.64	-2.01	0.01	**sig**	GRIA1	Glutamate receptor 1
TRINITY_DN4682_c0_g3	663.31	-1.15	0.01	**sig**	608.10	-0.52	0.20		171.47	-1.41	0.00	**sig**	GRIA2	Glutamate receptor 2
TRINITY_DN1604_c0_g2	75.08	-0.37	0.25		0.14	-0.71	0.04	**sig**	92.07	-1.33	0.00	**sig**	GRIA3	Glutamate receptor 3
TRINITY_DN26238_c0_g1	51.69	-0.75	0.03	**sig**	170.99	-0.74	0.02	**sig**	64.96	-1.50	0.00	**sig**	GRIA4	Glutamate receptor 4
TRINITY_DN3531_c0_g2	205.78	-0.60	0.05	**sig**	253.84	-0.31	0.18		257.92	0.43	0.51		GRID2	Glutamate receptor ionotropic, delta-2
TRINITY_DN4268_c1_g2	82.69	0.46	0.03	**sig**	141.97	0.23	0.48		25.48	-1.54	0.00	**sig**	GRIK1	Glutamate receptor ionotropic, kainate 1
TRINITY_DN3279_c0_g1	416.32	-0.17	0.26		0.51	0.00	0.99		890.05	0.91	0.00	**sig**	GRIK2	Glutamate receptor ionotropic, kainate 2
TRINITY_DN1892_c0_g1	110.69	-0.73	0.18		0.00	-0.29	0.23		69.90	-1.46	0.00	**sig**	GRIK3	Glutamate receptor ionotropic, kainate 3
TRINITY_DN8709_c0_g1	28.55	-0.96	0.04	**sig**	--	-0.43	--		7.66	-2.50	0.00		GRIK4	Glutamate receptor ionotropic, kainate 4

TRINITY_DN3560_c0_g1	389.61	-0.49	0.01	**sig**	683.77	0.07	0.86		803.02	0.55	0.25		GRM1	Metabotropic glutamate receptor 1
TRINITY_DN43867_c0_g1	2592.66	0.54	0.13		315.81	0.13	0.83		148.20	-1.61	0.03	**sig**	GRM3	Metabotropic glutamate receptor 3
TRINITY_DN3936_c1_g2	58.49	-0.32	0.33		145.94	0.02	0.98		19.72	-2.07	0.00	**sig**	GRM4	Metabotropic glutamate receptor 4
TRINITY_DN3560_c0_g2	1468.09	-0.55	0.01	**sig**	175.31	0.52	0.49		350.79	1.19	0.02	**sig**	GRM5	Metabotropic glutamate receptor 5
TRINITY_DN6478_c0_g1	510.39	0.03	0.89		505.70	0.10	0.58		435.66	0.79	0.01	**sig**	GRM7	Metabotropic glutamate receptor 7
TRINITY_DN879_c0_g2	91.23	-0.41	0.11		166.71	-0.28	0.31		109.16	-0.57	0.02	**sig**	KAPCA	cAMP-dependent protein kinase catalytic subunit alpha
TRINITY_DN30763_c1_g1	1675.92	-1.34	0.01	**sig**	640.61	-0.56	0.04	**sig**	456.11	-0.66	0.04	**sig**	KC2D2	Calcium/calmodulin-dependent protein kinase type II delta 2 chain
TRINITY_DN2644_c0_g1	491.22	-0.38	0.17		169.95	-0.06	0.87		86.85	-1.58	0.04	**sig**	KCC1D	Calcium/calmodulin-dependent protein kinase type 1D
TRINITY_DN1556_c1_g3	46.28	0.76	0.13		388.15	-0.04	0.93		175.79	-1.45	0.00	**sig**	KCC1G	Calcium/calmodulin-dependent protein kinase type 1G
TRINITY_DN2603_c2_g1	354.07	-0.78	0.01	**sig**	420.08	-0.20	0.37		373.08	0.20	0.68		KCC2A	Gamma-aminobutyric acid receptor subunit beta-3
TRINITY_DN1138_c0_g1	383.23	-0.77	0.03	**sig**	611.97	-0.48	0.14		777.07	0.34	0.52		NAC1	Voltage-dependent P/Q-type calcium channel subunit alpha-1A
TRINITY_DN14214_c0_g1	289.18	-1.00	0.01	**sig**	158.12	-0.44	0.24		469.01	-0.74	0.03	**sig**	NBEA	Glucocorticoid receptor
TRINITY_DN154_c0_g3	18.95	-0.50	--		50.90	-0.33	0.35		25.65	-1.32	0.03	**sig**	NMD3B	Glutamate receptor ionotropic, NMDA 3B
TRINITY_DN3279_c0_g4	229.15	-1.30	0.01	**sig**	59.98	-0.69	0.12		12.98	-2.13	0.00	**sig**	NMDE1	Glutamate receptor ionotropic, NMDA 2A
TRINITY_DN10257_c0_g1	589.83	-1.22	0.00	**sig**	152.56	-0.56	0.06		36.47	-1.64	0.00	**sig**	NMDE2	Glutamate receptor ionotropic, NMDA 2B
TRINITY_DN14421_c0_g2	33.15	-0.75	0.05	**sig**	79.49	-0.92	0.02	**sig**	21.24	-2.21	0.00	**sig**	NMDE4	Glutamate receptor ionotropic, NMDA 2D
TRINITY_DN3191_c0_g1	5200.41	-0.46	0.03	**sig**	3053.03	-0.05	0.84		599.03	-1.32	0.00	**sig**	NMDZ1	Glutamate receptor ionotropic, NMDA 1
TRINITY_DN10281_c0_g1	90.75	-0.08	0.83		117.35	0.40	0.22		101.98	1.02	0.00	**sig**	S6A13	Sodium- and chloride-dependent GABA transporter 2
TRINITY_DN196_c0_g1	541.72	-1.00	0.00	**sig**	421.41	-0.61	0.22		294.00	-0.65	0.07		SV2A	Synaptic vesicle glycoprotein 2A
TRINITY_DN3892_c0_g1	135.03	-0.96	0.01	**sig**	215.15	-0.55	0.04	**sig**	340.43	0.12	0.82		SV2C	Synaptic vesicle glycoprotein 2C
TRINITY_DN18175_c0_g3	104.22	0.38	0.51		0.00	0.00	0.00		19.11	-2.54	0.03	**sig**	VGL2B	Vesicular glutamate transporter 2.2

Table S14. Differentially expressed genes and Pituitary hormone genes (*) in the three brain regions of *A. leucosternon* during the interaction treatment. Column "SeqName" represents the transcript sequence obtained with their respective Trinity ID. Grouped columns by "Forebrain", "Midbrain" and "Hindbrain" contain the Differential expression analysis (DESeq2) results for each region where the values of log2fold changes are represented by positive (upregulated expression) or negative (downregulated expression) values, and its significance highlighted in **bold** (padj <0.05). Grouped columns by "Gene Annotation", represent the gene annotation for each transcript (Swissprot-blast), their respective Gene symbol and its full name

	**Forebrain**				**Midbrain**				**Hindbrain**				**Gene Annotation**	
**SeqName**	**Base Mean**	**log2 Fold Change**	**padj**	**SIG**	**Base Mean**	**log2 Fold Change**	**padj**	**SIG**	**Base Mean**	**log2 Fold Change**	**padj**	**SIG**	**Gene symbol**	**Gene name**
TRINITY_DN3769_c0_g1	19198.32	-9.92	0.00	**sig**	10.27	3.73	0.75		21.40	-2.66	--		POMC	Pro-opiomelanocortin*
TRINITY_DN44506_c0_g1	6195.94	-12.48	0.00	**sig**	8.92	1.75	0.95		8.23	-1.06	--		GLHA	Glycoprotein hormones alpha chain*
TRINITY_DN14466_c0_g1	112.15	-5.90	0.05	**sig**	9.68	-0.60	0.99		2.17	0.00	--		GON3	Progonadoliberin-3*
TRINITY_DN8623_c0_g1	419.68	-9.94	0.00	**sig**	1.47	-0.10	--		0.00	0.00	--		GTHB1	Gonadotropin subunit beta-1*
TRINITY_DN18356_c0_g1	169.44	-10.62	0.00	**sig**	0.00	0.00	--		0.56	0.00	--		GTHB2	Gonadotropin subunit beta-2*
TRINITY_DN4307_c0_g1	6653.92	-15.91	0.00	**sig**	10.75	3.01	--		1.07	-1.74	--		PRL	Prolactin*
TRINITY_DN16637_c0_g1	395.52	-1.37	0.00	**sig**	4.44	-1.10	--		86.45	-0.53	0.75		SMS1	Somatostatin-1*
TRINITY_DN8324_c0_g1	2914.80	-13.24	0.00	**sig**	0.15	0.00	--		1.10	-2.92	--		SOML2	Somatolactin-2*
TRINITY_DN8994_c0_g1	40598.07	-18.52	0.00	**sig**	21.87	3.12	--		5.24	-1.38	--		SOMA	Somatotropin*
TRINITY_DN14018_c0_g1	853.38	-10.59	0.00	**sig**	1.49	0.40	--		3.97	0.00	--		TSHB	Thyrotropin subunit beta*
TRINITY_DN6745_c0_g1	25.13	2.22	0.01	**sig**	71.84	1.36	0.32		53.14	1.56	0.07		CHP2	Calcineurin B homologous protein 2
TRINITY_DN4695_c1_g1	39.92	1.03	0.04	**sig**	94.89	1.29	0.32		81.10	1.05	0.19		FCRL5	Fc receptor-like protein 5
TRINITY_DN10330_c0_g1	293.47	0.75	0.00	**sig**	226.74	0.55	0.66		238.64	0.23	0.71		PTPRF	Receptor-type tyrosine-protein phosphatase F
TRINITY_DN14252_c0_g1	271.75	0.69	0.01	**sig**	95.64	4.80	--		92.32	0.82	--		ADA1B	Alpha-1B adrenergic receptor
TRINITY_DN57184_c0_g1	219.88	-7.98	0.00	**sig**	1341.10	-0.45	0.65		9124.53	0.01	0.99		GRID2	Glutamate receptor ionotropic, delta-2
TRINITY_DN5065_c0_g1	265.54	-0.60	0.02	**sig**	988.91	-0.48	0.38		831.72	0.04	0.94		DCE1	Glutamate decarboxylase 1
